# Improving analysis of transcription factor binding sites within ChIP-Seq data based on topological motif enrichment

**DOI:** 10.1186/1471-2164-15-472

**Published:** 2014-06-13

**Authors:** Rebecca Worsley Hunt, Anthony Mathelier, Luis del Peso, Wyeth W Wasserman

**Affiliations:** Bioinformatics Graduate Program, University of British Columbia, Vancouver, BC Canada; Centre for Molecular Medicine and Therapeutics, Child and Family Research Institute, Department of Medical Genetics, University of British Columbia, Vancouver, BC Canada; Biochemistry, Universidad Autónoma de Madrid, Madrid, 28029 Spain; Centre for Molecular Medicine and Therapeutics, 950 W.28th Avenue, Vancouver, BC V5Z 4H4 Canada

**Keywords:** Chromatin immunoprecipitation, ChIP-Seq, Motif prediction, Over-representation analysis, Regulation, Sequence analysis, Transcription factor, Transcription factor binding site, Visualization

## Abstract

**Background:**

Chromatin immunoprecipitation (ChIP) coupled to high-throughput sequencing (ChIP-Seq) techniques can reveal DNA regions bound by transcription factors (TF). Analysis of the ChIP-Seq regions is now a central component in gene regulation studies. The need remains strong for methods to improve the interpretation of ChIP-Seq data and the study of specific TF binding sites (TFBS).

**Results:**

We introduce a set of methods to improve the interpretation of ChIP-Seq data, including the inference of mediating TFs based on TFBS motif over-representation analysis and the subsequent study of spatial distribution of TFBSs. TFBS over-representation analysis applied to ChIP-Seq data is used to detect which TFBSs arise more frequently than expected by chance. Visualization of over-representation analysis results with new composition-bias plots reveals systematic bias in over-representation scores. We introduce the BiasAway background generating software to resolve the problem. A heuristic procedure based on topological motif enrichment relative to the ChIP-Seq peaks’ local maximums highlights peaks likely to be directly bound by a TF of interest. The results suggest that on average two-thirds of a ChIP-Seq dataset’s peaks are bound by the ChIP’d TF; the origin of the remaining peaks remaining undetermined. Additional visualization methods allow for the study of both inter-TFBS spatial relationships and motif-flanking sequence properties, as demonstrated in case studies for TBP and ZNF143/THAP11.

**Conclusions:**

Topological properties of TFBS within ChIP-Seq datasets can be harnessed to better interpret regulatory sequences. Using GC content corrected TFBS over-representation analysis, combined with visualization techniques and analysis of the topological distribution of TFBS, we can distinguish peaks likely to be directly bound by a TF. The new methods will empower researchers for exploration of gene regulation and TF binding.

**Electronic supplementary material:**

The online version of this article (doi:10.1186/1471-2164-15-472) contains supplementary material, which is available to authorized users.

## Background

Delineating the specific *cis*-regulatory elements governing gene transcription has been at the forefront of genome research. The landmark release of the ENCODE project findings supports the long-standing view that a greater portion of the human genome contributes to the regulation of gene activity than the ~2% encoding proteins. High-throughput chromatin immunoprecipitation (ChIP) analysis of protein-DNA interactions has been transformative, highlighting the genomic regions that contain regulatory elements, and thus reducing the computational search space for transcription factor binding sites (TFBSs) many fold. The coupling of ChIP to high-throughput sequencing (ChIP-Seq) is empowering researchers to investigate DNA binding transcription factors (TFs) and their regulation of the genome. We present here a set of convenient, practical visualization and bioinformatics approaches, which facilitate interpretation of ChIP-Seq TF binding data.

The bioinformatics foundations of generating ChIP-Seq data have been well explored. Given mapped DNA sequence reads from a ChIP experiment, “peak calling” software is applied to quantitatively delineate regions within which a greater frequency of mapped reads are observed than expected. The peak regions are reported as a pair of coordinates, ranging from 1 bp to >5000 bp wide, often accompanied by a score and the position at which the read frequency local maximum is observed. Within the peak regions, the coordinates of specific TF-DNA interactions can be computationally inferred, using a TFBS profile for the indicated TF. Such profiles, most commonly in the form of Position Frequency Matrices (PFMs), are available for a subset of TFs from databases like JASPAR [1] or HoCoMoCo [2], or can be computationally identified from the ChIP-Seq data using *de novo* motif discovery tools [3–6]. Analysis with a PFM converted to a weighted TFBS profile (Position Weight Matrix – PWM) yields a score that reflects the similarity of the sequence of interest to the modeled binding sites. Although ChIP-Seq data reduces the acknowledged specificity problem of detecting short (6-15 bp), degenerate motifs bound by a TF in the genome, the problem of TFBS prediction is not perfectly resolved as the ChIP-Seq peaks are often 20-fold or greater in length than the TFBS being searched for. As they become more widely used, higher resolution methods, such as ChIP-exo [7], are expected to reduce the difficulty.

A proportion of ChIP-Seq peaks may not contain a canonical TFBS for the ChIP’d TF above background expectation; a confounding property of the data that presumably arises from a combination of biological, experimental, and computational influences. While these regions may result from indirect interactions between the TF and the DNA, the multi-epitope specificity of polyclonal antibodies and the tendency for chromatin to shear at promoter regions [8, 9] may give rise to peaks not specific to the ChIP’d TF. The subset of peaks lacking the TF’s canonical motif is commonly treated as equivalent to the subset with motifs. The segregation of a ChIP-Seq dataset into the two classes could lead to insights into individual TFs mechanisms of regulation and reveal common properties of regions lacking TFBS. The analysis of specific TF bound regulatory regions and TFBSs from ChIP-Seq defined peak regions can be refined, and such improvement will consequently inform and improve our utilization of ChIP-Seq data across a spectrum of analyses.

In this report, we introduce a set of visualization methods and bioinformatics approaches to improve the study of TFBSs within ChIP-Seq regions, and demonstrate the application of these methods for the generation of new insights into regulatory sequences. We focus on three key challenges: known motif over-representation analysis, spatial visualization of TFBS positions, and determination of parameters for TFBS analysis. For over-representation analysis, we introduce the BiasAway tool to account for the non-random properties of regulatory sequences; such accounting has strongly informed the design of *de novo* motif discovery methods, but has been inadequately addressed for over-representation studies. We introduce a set of visualization approaches that reveal topological patterns of motif positions within ChIP-Seq data, helping to delineate the subset of peaks likely to be directly bound by the ChIP’d TF. The visualization methods directly inform the selection of parameters for motif prediction, a long-standing challenge in regulatory sequence analysis. Application of the procedure reveals that on average 61% of ChIP-Seq peak regions contain the canonical motif for the ChIP’d TF. The methods are applied to two cases related to TBP and ZNF143/THAP11. Access to the new methods and visualization approaches will provide the research community with improved capacity to analyze and interpret TF ChIP-Seq data.

## Results

### Composition studies reveal the influence of non-random properties of the metazoan genome on the interpretation of ChIP-Seq data

The non-random properties of genome nucleotide sequence composition have been the subject of extensive investigation over decades. Key to the study of regulatory sequences, are observations that promoters and other open chromatin tend to have higher GC content, and that the observed composition has a wide distribution. We evaluated the relationship between mononucleotide composition and TF ChIP-Seq data (see Additional file 1: Text S1). We found that each TF exhibits a range of nucleotide composition environments in which it binds. The GC content distributions differ between TFs profiled in the same cells and between different cell types profiled for the same TF (Additional file 2: Figure S1). There is a clear relationship between the GC content of the peak regions and the multiplicity of TFBSs (Additional file 1: Text S1, Additional file 3: Figure S2).

### Consequences of biased composition for TFBS motif over-representation analyses

It is a central practice in the analysis of regulatory regions to evaluate the frequency of predicted TFBSs in the regions of interest. This type of analysis depends on comparing predicted motif frequencies against a reference background. Such analysis of TFBS over-representation could be influenced by the compositional properties of the peaks and the background sequences. While accounting for background composition properties has been explored for *de novo* pattern discovery, the best approaches for and impact of composition correction on over-representation analysis of known motifs have not been resolved.

#### Visualizing composition bias in TFBS *over-representation*analyses

**Figure 1:**
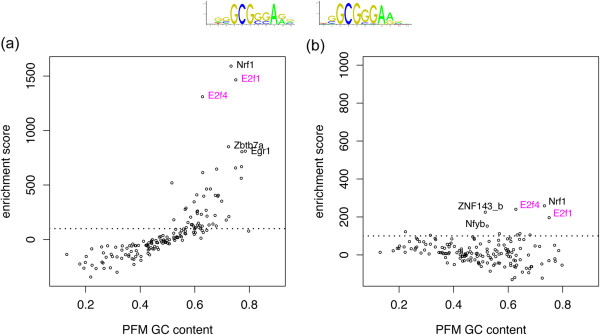
**Composition-bias plots reveal systematic TF PFM nucleotide content bias in motif over-representation analysis.** Foreground data was obtained from an E2F1 ChIP-Seq study and processed using the oPOSSUM TFBS over-representation analysis software. Each plot presents a motif over-representation score (y-axis) relative to the GC content of the PFMs (x-axis). The over-representation scores reflect the difference between the frequency of motifs in the foreground compared to a background (the background differs between panels). The names of the 5 top ranked PWMs are displayed on the plot. The dotted line at over-representation score 100 is an arbitrarily placed visual point of reference. The sequence logos represent the binding models for E2F1 and E2F4 respectively. **(a)** Background composed of randomly selected mappable genome sequences. **(b)** Background generated, using BiasAway, with a GC composition matched to the ChIP-Seq sequences and drawn from the set of mappable sequences.

To assess composition corrective measures for TFBS over-representation analysis, we generated a visualization method, which we term Composition-Bias plots (CB-plots). The CB-plots are a method for alerting a researcher to the impact of composition on the reported TFBS over-representation results, and are generally more informative than a ranked list of TFs and motif over-representation scores. A CB-plot presents the average GC content of a TF’s binding profile (the PFM) on the x-axis and the over-representation score of the TF’s predicted binding sites on the y-axis. If an unsuitable background has been used in the over-representation analysis, the CB-plot distribution of motif over-representation scores will reveal a bias toward whichever end of the nucleotide composition spectrum (GC-rich or AT-rich) that the target sequences dominate relative to the background. Figure 1 illustrates the CB-plots and provides examples of over-representation results from an E2F1 ChIP-Seq dataset. Figure 1a presents results that need to be corrected for over-representation score bias, as outlined in the next section, while Figure 1b presents the end results after correction.

#### The impact of background composition selection on TFBS *over-representation*results

For many datasets the calculation of meaningful over-representation scores requires correction of bias. We assessed approaches to create and/or retrieve a background matched to a set of target sequences’ compositional properties in order to resolve the enrichment scoring bias engendered by compositional extremes of some ChIP-Seq datasets. For evaluation purposes we retained the naïve background model of random genomic sequences in our assessment. A second background was generated by a 3rd order Markov model (RSAT package [10]). We implemented a background sequence generator, BiasAway, to generate 6 additional background models; these backgrounds derived from mono- and di- nucleotide shuffled sequences, and genomic sequences matched to the GC content of the target data (see Additional file 1: Text S1 for details). We included one last set of backgrounds generated by “known motif” over-representation analysis feature of HOMER 2 [11]. HOMER 2 is the only software of which we are aware that uses GC composition matched backgrounds for TFBS over-representation analysis. These backgrounds were then evaluated against 43 human TF ChIP-Seq datasets, with 166 PWMs (JASPAR 4.0_alpha development database [1]) *via* the oPOSSUM 3.0 TFBS over-representation software [12]. Four backgrounds were re-evaluated for platform-independence of both bias and bias correction with the ASAP tool (see Additional file 1: Text S1 and Additional file 4: Figure S3).

**Figure 2:**
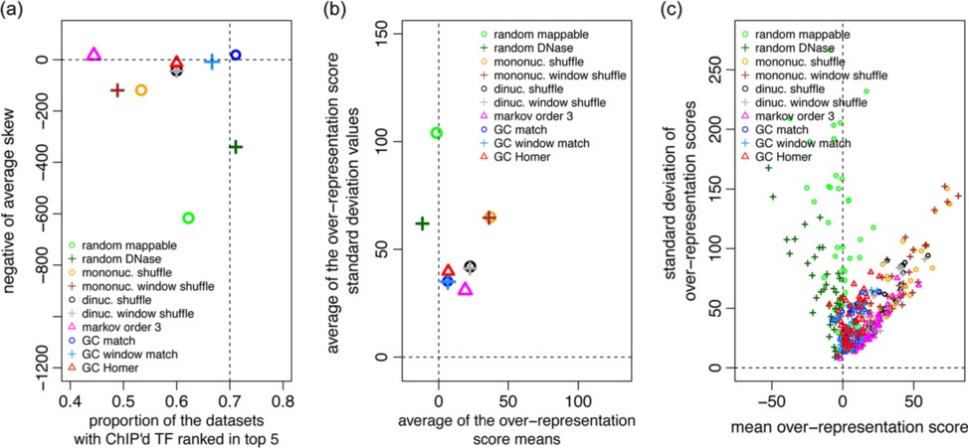
**Background sequence selection impacts motif over-representation analyses. (a)** For each background, the fraction of the 43 analyses that reported the ChIP’d TF in the top 5 enriched PWMs from a particular background (x-axis) is plotted against the average skew of the over-representation results for each background’s 43 analyses. Skew is the negative slope of the line fitted to the over-representation scores *versus* PFM GC content (*i.e.* values as displayed in Figure 1). The ideal is to have a large x-axis value (vertical dashed line) and an average skew of zero (horizontal dashed line). **(b)** and **(c)** summarize the standard deviation (y-axis) and mean (x-axis) of the ‘non-outlier’ oPOSSUM over-representation scores for 10 backgrounds against each of 43 ChIP-Seq datasets, where panel **(b)** displays the average value for each background across the 43 datasets and panel **(c)** displays the individual value of 430 analyses. The ideal result would be situated at the origin (the intersection of the dashed lines). For all panels each of the 10 backgrounds tested is denoted as a single colour: Light green circle – randomly chosen background from the dataset of mappable sequences, dark green cross – randomly chosen background from the dataset of DNase accessible sequences, orange circle – mononucleotide shuffled background, brown cross – mononucleotide shuffled background within a sliding window, black circle – dinucleotide shuffled background, gray cross – dinucleotide shuffled background within a sliding window, magenta triangle – 3rd order Markov model generated background sequences, blue circle – background selected from the mappable sequences dataset to match the GC composition of the target sequences, light blue cross – background selected from the mappable sequences dataset to match the distribution of GC composition in sliding windows of the target sequences, and red triangle – GC background from HOMER 2.

We established several measures, based on the ideal expectation of four properties pertaining to the results of TFBS over-representation analyses. In essence, we expect to see few outlying over-representation scores for TF binding models, one of which should be for the ChIP’d TF’s binding model, with the majority of binding models scoring close to 0. We summarize the results for each of the alternative background models using these measures in Figure 2 and Additional file 5: Figure S4.

We summarize the background evaluations on 201 bp sequences here, with further details available in Additional file 1: Text S1 for both 201 bp and 401 bp sequences. Additional file 6: Table S1 lists the rank of the ChIP’d TF for each analysis, and Additional file 7: Figure S5 provides the CB-plots of E2F1 for six background analyses. A naïve background of randomly selected sequences resulted in a systematic bias, or skew, of over-representation scores towards GC-rich binding sites for some datasets. The mononucleotide backgrounds were not as favourable as the dinucleotide shuffled backgrounds, with or without the sliding window. While neither suffered from bias towards GC-rich binding sites, the dinucleotide shuffled backgrounds produced results with over-representation scores closer to zero and predicted the ChIP’d TF’s motif in the top 5 results of more than 60% of the datasets. The 3rd order Markov model performed as well as the dinucleotide background for all measures except that of capturing the ChIP’d TF’s motif in the top 5 results. The best performing background was genomic sequences selected to match the GC nucleotide distribution of the target sequences. The BiasAway GC nucleotide background and HOMER generated backgrounds performed comparably for most measures, with the exception that the BiasAway background resulted in an 11 percentage point improvement for the inclusion of the ChIP’d TF binding model as one of the top 5 over-represented.

The CB-plots have been incorporated into the oPOSSUM over-representation analysis software, and BiasAway is available as open-source code posted on GitHub: https://github.com/wassermanlab/BiasAway/archive/noRPY.zip, and is being incorporated into the oPOSSUM 3.0 web interface.

#### Assessing TFBS predictions within ChIP-Seq regions

Subsequent to motif over-representation analysis, attention turns to investigating the candidate TFBS binding profiles. We outline here a complementary set of enrichment assessment methods specifically oriented to providing such insights. We implement a heuristic algorithm for direct binding (HADB) to determine the subset of ChIP-Seq peaks with high confidence binding sites, based on TFBS enrichment proximal to the peakMax, and to derive a PWM-specific scoring threshold. As with the previous section, visualization is a key method for discerning the properties of the data. Within the R environment [13] we implemented a set of plotting methods (TFBS-landscape view, TFBS-bi-motif view, and Dinucleotide-environment view) for displaying the properties of the ChIP-Seq regions relative to the locations of predicted TFBS. The code and user-guide for the visualization methods and calculating the HADB thresholds are posted on GitHub: https://github.com/wassermanlab/TFBS_Visualization/archive/master.zip.

### TFBS-Landscape view

The TFBS-landscape view for ChIP-Seq data facilitates qualitative assessment of the non-random relationship between predicted motif scores (for the top scoring motif in each sequence) and the peakMax position. The information in the TFBS-landscape view is translatable into a quantitative assessment of TFBSs, as presented by the HADB algorithm below. Motif proximity to the peakMax has been demonstrated on 14 ChIP-Seq datasets by [14] and for 3 datasets across 11 peak calling algorithms [15]. The global tendency for ChIP-Seq data to yield motifs for the ChIP’d TF proximal to the peakMax is systematically confirmed here with a study of ~340 ChIP-Seq datasets for ~95 TFs (human and mouse).

**Figure 3:**
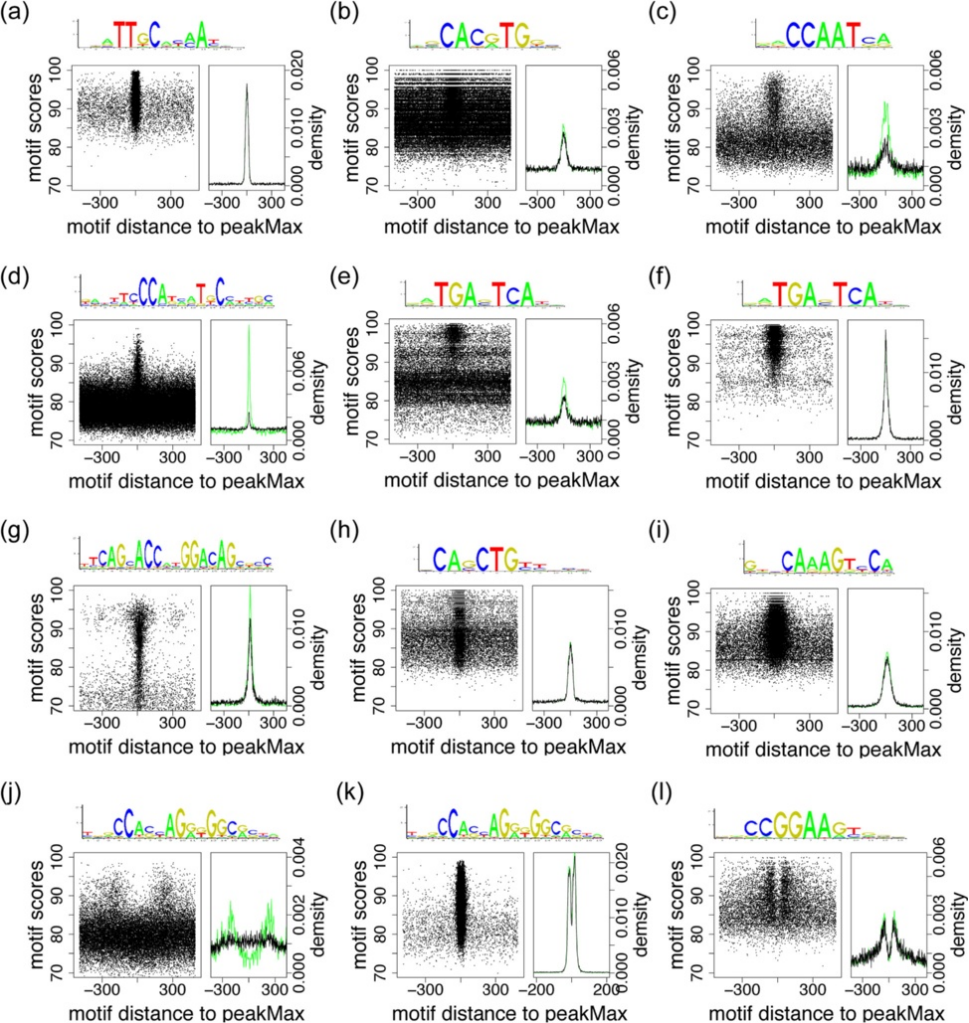
**TFBS-landscape views inform of motif enrichment and are diverse in shape and density.** A TFBS-landscape view consists of a plot (left) showing the location of the top scoring motif relative to the peakMax (x = 0) on the x-axis and the motif score on the y-axis; the right plot presents a 2 bp resolution density of motif distances to the peakMax (black) and 5 bp resolution for motifs with a motif score equal to or greater than 85 (green). All plots display some degree of enrichment at the peakMax and a lower limit on motif score enrichment. **(a)** C/EBPB motifs in a C/EBPB ChIP-Seq dataset. **(b)** C-MYC motifs are enriched at the peakMax of the C-MYC ChIP-Seq dataset, but many top-scoring motifs are randomly dispersed. **(c)** NFYA motifs in a NFYA ChIP-Seq dataset exhibit enrichment around the peakMax, with high scoring motifs distinct from the majority of scores in the background regions **(d)** ZNF143 motifs in a ZNF143 ChIP-Seq dataset have low enrichment but some high scoring motifs are distinct from the background. **(e)** and **(f)** present JUN motif enrichment in two JUN datasets from different cell types with distinct background motif densities. **(g)** The REST motif is strongly enriched in a REST ChIP-Seq dataset across a large motif score range at the peakMax with a low density of background motifs. **(h)** MYOD motif enrichment at the peakMax of MYOD ChIP-Seq data. **(i)** HNF4A motif enriched proximal to the peakMax of HNF4A ChIP-Seq data. **(j)**, **(k)** and **(l)** present motif enrichment for a TF that was not the ChIP’d target: **(j)** CTCF motifs are slightly enriched offset from the peakMax of H3k4me3 ChIP-Seq. **(k)** CTCF motifs are enriched offset from the peakMax in RAD21::cohesin ChIP-Seq data. **(l)** ELK4 motifs in a NELFE ChIP-Seq dataset show an enrichment offset from the peakMax.

In a TFBS-landscape view, the left plot displays the distance of the maximum scoring TFBS to the peakMax for each sequence on the x-axis (the peakMax is x = 0), and the PWM predicted TFBS score on the y-axis (Figure 3). This plot conveys both the quality of the motifs observed, and allows users to detect enrichment in a threshold-independent manner. The right plot presents the density of top-scoring predicted motifs (y-axis) versus the distance from the peakMax (x-axis), similar to plots presented by CentriMo (MEME suite [14]) and RSAT::peak-motifs tools [6]. The right side plots include two lines: a 2 bp resolution density of all peaks’ motifs, and a 5 bp resolution density of the subset of peaks containing a motif with score equal to or greater than 85 to capture cases with low enrichment of strong scoring motifs. As shown in Figure 3a for the TF C/EBPB, a ChIP-Seq experiment can show a strong enrichment for the ChIP’d TFs binding motifs. Plots representing the wide variety of enrichment characteristics are displayed in Figures 3a–l. The unique shapes displayed in Figure 3 derive from a combination of properties for the presented TF’s binding motifs such as width of the binding site, and the presence of other motifs enriched at the peakMax (see Additional file 1: Text S1 for further detail and discussion).

### Using the HADB algorithm to distinguish direct binding and for the selection of PWM motif score thresholds

**Figure 4:**
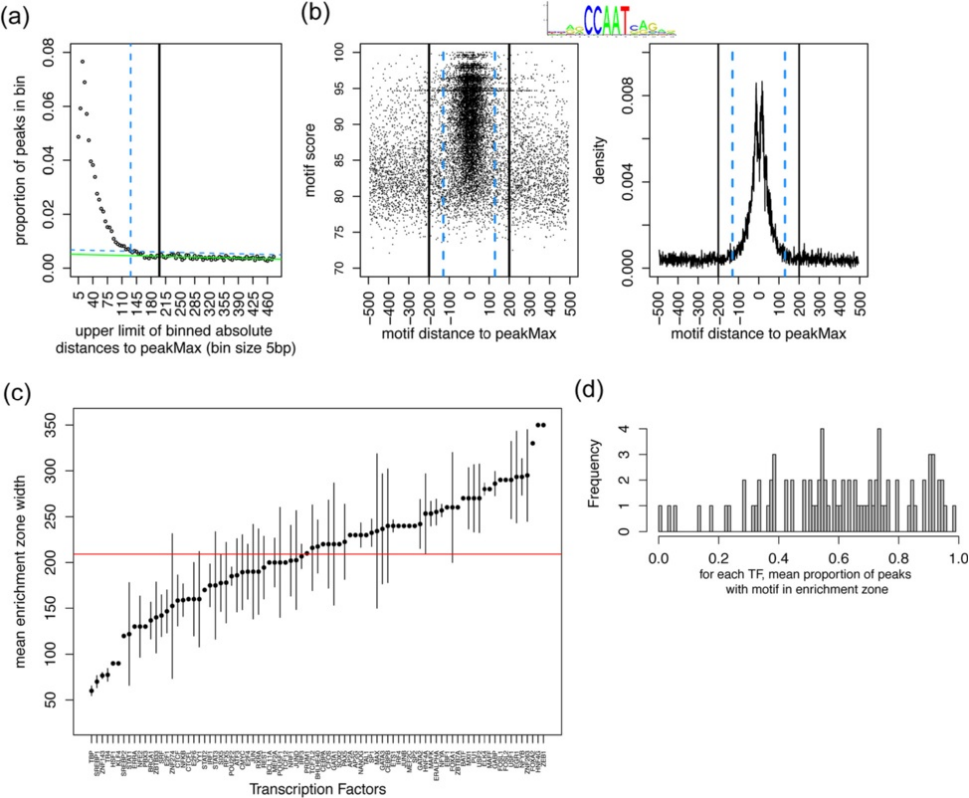
**Defining the TFBS zone of enrichment around the peakMax. (a)** A visual depiction of the enrichment zone determined with a heuristic procedure, as described in methods. The x-axis presents the upper limit of each 5 bp bin, and the bins are the absolute distance of a motif from the peakMax. The y-axis shows the proportion of peaks from the dataset with a motif in a 5 bp bin. The horizontal green line is fit to the distal background bins, and the horizontal line in blue is the allowance line (see methods). The blue vertical dashed line indicates where the proportion of peaks in a non-background bin approaches the allowance line without falling below it – this line is the heuristic distance threshold for motif enrichment around the peakMax. **(b)** The NFYB sequence logo and the TFBS-landscape view for NFYB motifs in NFYB ChIP-Seq data. The heuristic enrichment zone is between the blue vertical dashed lines. The black vertical lines indicate the beginning of the distal background region (spanning 200-500 bp from the peakMax). **(c)** The width of the motif enrichment zone (y-axis) for human ChIP-Seq datasets (x-axis); multiple datasets for a TF were averaged to obtain one enrichment zone value per TF. Vertical bars are the average differences between all of the enrichment zone widths for a TF. The red horizontal line is the mean. **(d)** The proportion of peaks within the enrichment zone for a TF’s set of ChIP-Seq datasets were averaged. The x-axis provides, for each of 85 TFs, the mean proportion of peaks with a motif scoring above the motif score threshold and located within the zone of enrichment (mean 0.60, median 0.61).

The TFBS-landscape plots present a finite zone of non-random TFBS enrichment around the peakMax for the ChIP’d TF. Quantitative analysis of the motif densities for a given TF can delineate the width of sequence proximal to the peakMax that is enriched for the TF’s motif above chance expectation. The enrichment zone provides a region of confidence for predicted TFBSs and thus provides a rational focus for downstream analyses. As shown in Figure 4a-b, the HADB procedure (see methods) determines the distance from the peakMax to the point where the frequency of the top scoring motif approaches the distal flank motif frequency (distal = 250-500 bp from the peakMax). Based on the pool of human ChIP-Seq datasets and focusing on the top ranked motif of each sequence, the width of enrichment around the peakMax ranges from ~50-350 bp with an average of 209 ± 34 bp or 194 ± 10 bp for human and mouse respectively (mean width ± mean of width differences; Figure 4c and Additional file 8: Figure S6a). Assessing enrichment with the inclusion of lower ranked motifs did not change the width of the observed enrichment zone.

Deciding upon an appropriate minimum scoring threshold for PWM-based analysis is an ongoing challenge, as each TF has unique characteristics. Quantitative analysis of the peak TF motif densities relative to chance expectation can delineate a suitable threshold for motif scores for a given PWM. We set the motif score threshold as the lowest motif score for which the count of motifs within the heuristic enrichment zone consistently exceeded the count of motif scores in comparably sized background regions (see methods; Additional file 9: Figure S7a-b). The motif score threshold does not vary greatly for an individual PWM across multiple datasets. The average relative score for each of the human and mouse datasets was 82 ± 1 (mean score ± mean difference between scores; Additional file 9: Figure S7c-d). A motif score threshold specific to each TF PWM is important for downstream analyses such as the study of TFBS altering mutations. The PWM score thresholds for the studied TF binding profiles are provided in Additional file 10.

We used the heuristic boundaries of enrichment and motif score threshold for each dataset to estimate the proportion of peaks that contain at least one TFBS for the ChIP’d TF within the bounds of the thresholds. We found that on average, for the datasets studied, ~61% of a ChIP-Seq dataset contains the ChIP’d TF’s canonical motif that is both within the peakMax enrichment zone and greater than the motif score enrichment threshold (Figure 4d – human mean 61%, and Additional file 8: Figure S6b – mouse mean 65%). The source of the remaining ~40% of peaks may be from such factors as indirect binding, shearing properties of open chromatin, peak calling errors, or antibody properties.

### Regions predicted by HADB to directly bind the ChIP’d TF are more likely to replicate between experiments

When available, replicate experiments are a useful validation of regions ChIP’d by a TF. While many experiments lack replicates, ~100 ENCODE datasets included replicates which we used to evaluate whether the HADB method was differentiating between peaks that co-occur between replicates (peakMax within 500 bp) *versus* those peaks that occur in only one of the replicates (see methods). We found that the set of peaks with the ChIP’d TFs motif central to the peakMax were significantly enriched for replicated peaks (92% of datasets produced a Fisher exact test one tailed p-value <0.001 (91% produced scores <1e-09)) (see Additional file 1: Text S1 for similar results with different peakMax separation parameter settings)).

### Regions predicted by HADB to directly bind the ChIP’d TF are enriched for GO terms related to the TF’s key biological process

To assess the functional enrichment of peaks defined by the HADB method, we performed GO enrichment analyses, using the GREAT software [16]. As GO enrichment analysis for TFBSs is somewhat problematic due to the diversity of processes a TF may regulate and the proximity of TFBS to the genes they regulate, we selected two TFs known to be highly specific for a biological process: SRF, a master regulator of the actin cytoskeleton and contractile processes [17], and NFE2L2, a key regulator of oxidative stress response [18]. We submitted peaks from the whole ChIP-Seq dataset, peaks from the subset of regions inferred by HABD to be directly bound by the ChIP’d TF, and those not inferred to be directly bound. GREAT analyses reported an enrichment of terms for the expected biological processes for SRF and NFE2L2 only in the subset of peaks with inferred direct binding of the ChIP’d TF (see Additional file 1: Text S1, Additional file 11: Figure S8 and Additional file 12: Figure S9). The results highlight how the use of the HADB method can enhance the interpretation of ChIP-Seq data.

### Complementary visualization methods for spatial ChIP-Seq patterns: TFBS bi-motif view and Dinucleotide-environment view

We present two visualization methods that further empower investigation of spatial patterns in ChIP-Seq data that build upon the foundation of peakMax-proximal enrichment defined by HADB.

### TFBS-bi-motif view

**Figure 5:**
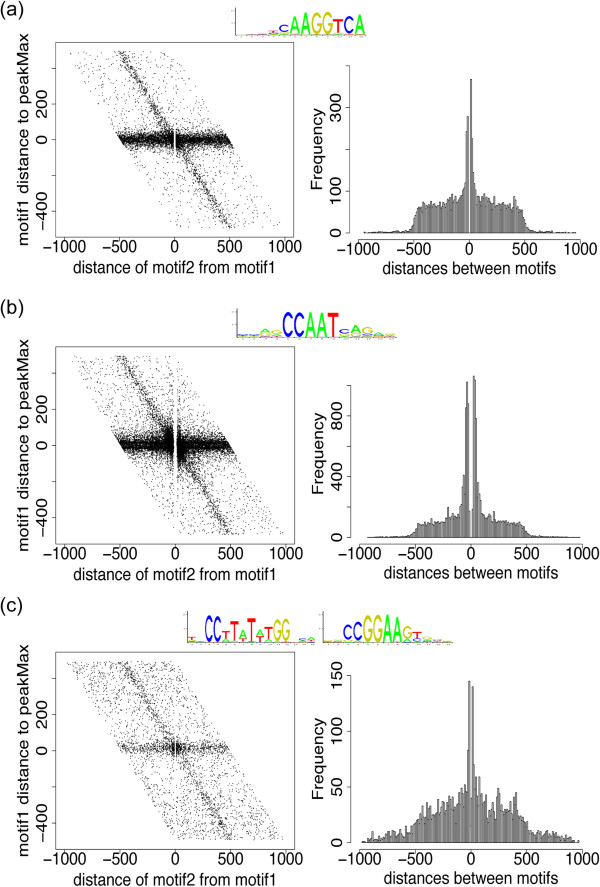
**TFBS-bi-motif view for visualization of motif spatial arrangements.** The left plot of a TFBS-bi-motif view presents the distance of the primary motif to the peakMax of each sequence on the y-axis, and the distance of a second motif relative to the primary motif on the x-axis. A band of enrichment at y = 0 indicates enrichment near the peakMax for the primary motif, while a diagonal band of enrichment (with a negative slope) indicates enrichment near the peakMax for the second motif. The diagonal limits of the plot arise from the uniform length of the sequences (here 1001 bp). The right plot is a histogram of the distances between the two motifs. The gap in both plots results from the exclusion of overlapping motifs. **(a)** ESRRB motifs in an ESRRB ChIP-Seq data set. The top-scoring ESRRB motif is the primary motif, and the second-best motif is the second motif. **(b)** NFYB motifs in a NFYB ChIP-Seq data set. The top-scoring NFYB motif is the primary motif, and the second-best motif is the second motif. **(c)** SRF ChIP-Seq dataset. The SRF top-scoring motif is the primary motif, and the ELK4 top-scoring motif is the second motif.

The prior TFBS-Landscape view focused on the ChIP’d TF in isolation. A substantial body of literature focuses on cooperativity between TFBS, both in homotypic and heterotypic forms [19–21]. In developing the HADB analyses we had observed that in some cases a second motif for the ChIP’d TF was enriched proximal to the peakMax, which motivated the creation of a TFBS-bi-motif view (examples are presented in Figure 5). The view allows qualitative assessment of both spatial relationships between the two motifs and their distance from the peakMax. If the first motif is enriched around the peakMax there is a horizontal band of enrichment along y = 0. If the 2nd motif is enriched around the peakMax there is a negatively sloped band of enrichment on a diagonal. Where the origin displays increased enrichment relative to the horizontal band (y = 0), there is an enrichment of both motifs at the peakMax. The diagonal limits of the plot arise from the uniform length of the peak regions analyzed. The right plot in the TFBS-bi-motif view presents a histogram of the distances between the two motifs. The proximity of two motifs can indicate potential interactions or relationships.

Three examples of bi-motif views are presented in Figure 5. Homotypic clustering is observed for ESRRB and NFYB. The NFYB clustering is consistent with published findings for the NF-Y complex [22], while the ESRRB observation suggests that properties observed for ESRRG may extend to other members of the TF family [23]. A third plot for SRF and ELK4 (also called ‘SRF accessory protein’), using SRF ChIP-Seq data, presents heterotypic binding site results, consistent with known interactions between these TFs [24].

### Dinucleotide-environment view

**Figure 6:**
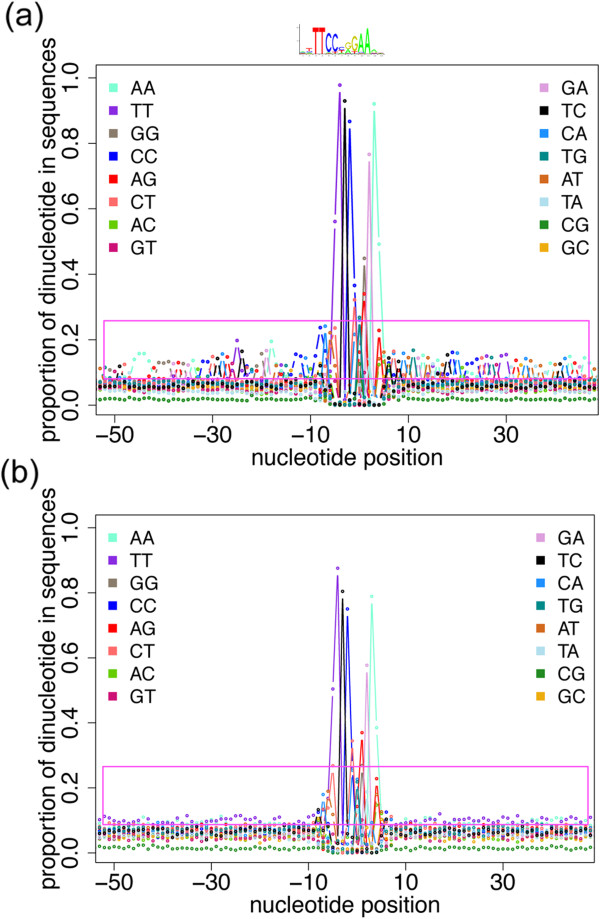
**Dinucleotide-environment view plots the dinucleotide enrichment of the dataset around the motif of interest.** The x-axis shows the dinucleotide offset from the centre of the motif, and the y-axis is the proportion of the dinucleotide in the ChIP-Seq sequences. The STAT1 motif is the high frequency pattern in the centre of the plot. The sequence logo for STAT1 is above the high frequency pattern. **(a)** The subset of STAT1 ChIP-Seq peaks containing a STAT1 motif in the enrichment zone with a motif score greater than or equal to 85. The magenta box highlights the enrichment of dinucleotides in the flanking regions of STAT1 motifs proximal to the peakMax. **(b)** The subset of STAT1 peaks with a motif outside the enrichment zone and a motif score of 85 or greater. The magenta box highlights the lack of dinucleotide enrichment in the flanking regions of motifs found distal to the peakMax.

Given the position of the motif of interest within a region, it can be informative to visualize the nucleotide composition properties in the flanking sequence. Dinucleotide sequence properties flanking TFBSs can be evaluated by aligning sequences at the TFBS (or a feature near the TFBSs, such as transcription start sites) and scoring the dinucleotide frequencies in the adjacent flanks. As shown in the Dinucleotide-environment view for an IFN-γ induced STAT1 TF ChIP-Seq experiment (Figure 6), there is increased nucleotide patterning in the sequences adjacent to the HADB-inferred directly bound motifs, compared to the peaks without peakMax-proximal motifs. Further analysis of the data reveals the presence of a common repeat sequence within a large subset of the peaks containing the highest scoring STAT1 motifs, consistent with literature which reports that STAT1 binding at MER41 repeat elements increases with IFN-y induction [25]. Further illustration of insights provided by the dinucleotide-environment view is provided in the case studies.

### An Applied Case Study of ChIP-Seq Analyses

To demonstrate how over-representation analysis and the visualization methods are complementary, and enhance the analysis of ChIP-Seq data, we applied the TFBS-landscape view and the Dinucleotide-environment view to two case studies. The first focuses on the TATA binding protein (TBP) in the MEL mouse cell line, while the second explores the sequence properties proximal to ZNF143 TFBSs.

**Figure 7:**
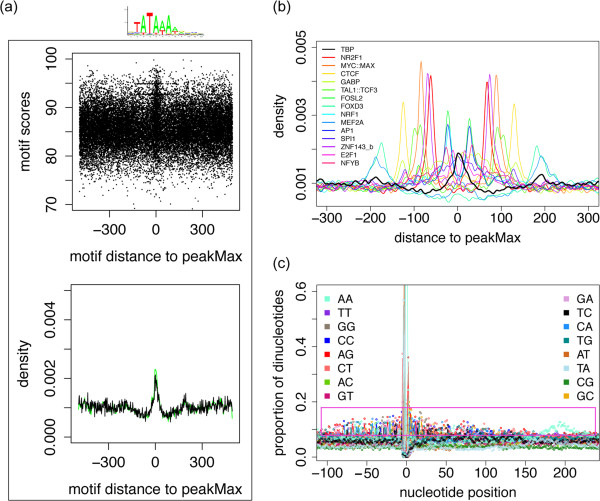
**Case study of TBP in the mouse MEL cell-line. (a)** TFBS-landscape view for TBP PWM on a TBP dataset. The top plot presents the top scoring motif distance to the peakMax (x-axis) and the motif relative score on the y-axis. The bottom plot presents a histogram of motif distances to the peakMax: black line – 2 bp resolution of the top scoring motif distance per peak; green line – 5 bp resolution of distances for the top scoring motifs with a score equal to or higher than 85. The sequence logo is for TBP. **(b)** TFBS-landscape view density plots for 15 PWM’s are overlaid on a single plot, for visualization purposes. The black line is the enrichment of the TBP motif, the coloured lines are NR2F1, MYC::MAX, CTCF, GABPA, TAL1::TCF3, FOSL2, FOXD3, NRF1, MEF2A, AP1, SPI1, ZNF143_b, E2F1, and NFYB motifs, as noted on the plot. **(c)** The Dinucleotide-environment view around the TBP motif. The x-axis is the location of the dinucleotide with respect to the TBP motif, and the y-axis is the fraction of sequences with the dinucleotide at a given position. The coloured lines each represent one of 16 dinucleotides, as specified in the plot legend. The magenta box highlights the dinucleotide enrichment in the regions flanking the TBP motif.

Employing both a motif over-representation analysis and TFBS-landscape view, we noted that the TBP ChIP-Seq experiment had a low occurrence of the canonical TBP TATA motif proximal to the peakMax (<12% of the dataset using the HADB method described above; Figure 7a). However, TFBS motif over-representation analysis also indicated a large number of secondary TFBSs are enriched. We investigated the reported enriched motifs using the TFBS-landscape view, and saw an unusual pattern emerge: spikes of lower scoring motif enrichment at distances from the peakMax specific to each tested TF (Additional file 13: Figure S10). As shown in Figure 7b, there are TF motif enrichment patterns up to 200 bp from the peakMax. A Dinucleotide-environment view, using the subset of peaks with a high scoring TATA motif, presented an enriched sequence pattern up to 100 bp either side of the TATA motif and an AA-rich sequence ~200 bp distant on the motif strand (Figure 7c). The pattern was subsequently identified to arise from enrichment of SINE elements (predominantly B2-B4). The percentage of peaks containing SINE elements is 14.1% in the TBP peaks, while in GC content matched controls or DNase accessible regions it drops to 5.2% and 5.6% respectively. While SINE elements are known to contribute to the formation of promoter regions for genes [26], the 14.1% fraction of the TBP ChIP-Seq sequences is striking.

**Figure 8:**
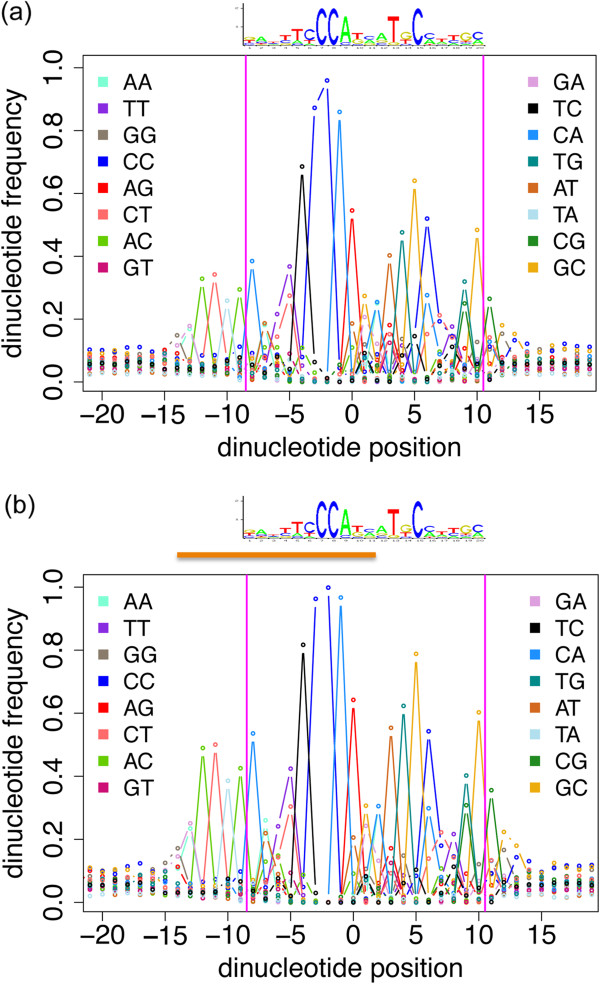
**Case study of ZNF143 DNA binding preferences.** The sequence logo presents the binding site characteristics of ZNF143. **(a)** Dinucleotide-environment view of ZNF143 ChIP-Seq repeat-masked regions aligned on motifs with a motif score of 85 or greater. The x-axis is the nucleotide position and the y-axis is the frequency of the dinucleotide. The coloured lines each represent one of 16 dinucleotides, as specified in the plot legend. The vertical magenta lines frame the positions of the sequence logo. **(b)** Dinucleotide-environment view of ZNF143 canonical motifs with a motif score of >85. The x-axis is the nucleotide position and the y-axis is the frequency of the dinucleotide. The coloured lines each represent one of 16 dinucleotides, as specified in the plot legend. The orange horizontal line above the plot indicates the overlapping THAP11 binding profile.

The seven zinc finger ZNF143 TF has been reported to bind to an ~18-21 bp nucleotide sequence [27, 28]. The ZNF143 ChIP-Seq dataset, like the TBP dataset, exhibits a low enrichment of the canonical ZNF143 motif around the peakMax (~5% of the dataset; Figure 3d). We extracted the subset of regions with a motif in the HADB enrichment zone, repeat-masked the sequences, and generated a Dinucleotide-environment view aligned on the top-scoring motif in each region (Figure 8a). This display reveals additional striking features not captured in the initial model, including a strong 5’ pattern outside the PWM-covered positions. This 5’ flank pattern is present in about 35% of the aligned sequences, and is not present in alignments of motifs outside of the HADB enrichment zone. A Dinucleotide-environment view of sequences with a high scoring (score >85) ZNF143 motif, reveals an increased enrichment of the 5’ flanking pattern from 35% to ~50% of the sequences (Figure 8b). A subsequent search of the literature revealed a recent study by Ngondo-Mbongo *et. al*. [29], showing that a 2nd protein, THAP11, binds to the extended flank seen in the Dinucleotide-environment view and half of the ZNF143 motif in a manner mutually exclusive to ZNF143.

## Discussion

We have introduced methods that allow researchers confronted with ChIP-seq data for TF binding to extract more insights into TFBS. The first challenge in such studies is often the identification of contributing TFs based on known motif over-representation analysis. Such methods can both support the quality of data based on the identification of the ChIP’d TF’s motif, as well as highlight additional TFs that may be cooperatively acting with the former. In this report we present two key components related to over-representation analysis. First, Composition-bias (CB) plots are introduced to display skew in the GC-content of the enriched motifs, a common occurrence that reflects the non-random composition properties of the ChIP-Seq regions recovered. Second, the BiasAway software tool is introduced to generate composition-matched background sequence sets that correct for the skew. Once relevant TF binding profiles are identified, the challenge shifts to the identification of reliable TFBS within the broader ChIP-Seq regions. Every ChIP-Seq dataset is a mixture of directly bound segments and regions that may reflect alternative influences. We introduce TFBS-landscape plots as a convenient form for the visualization of the topological distribution of TFBS within ChIP-Seq segments. Based on the topology, we present the HADB method for the selection of PWM thresholds for the selection of candidate TFBS consistent with direct binding events. The HADB approach provides a quantitative method for selecting PWM thresholds – an enduring challenge in regulatory sequence analysis. Systematic analysis over ~340 ChIP-Seq datasets indicates that an average of 61% of peaks are classified as directly bound. The biochemical, experimental, and/or computational influences that account for the remaining 39% of regions remain to be resolved in future investigation. In the third stage of analysis, spatial properties relative to the defined TFBS and peakMax are analyzed. Bi-motif plots allow the study of inter-TFBS spacing concurrent with HADB assessment. To gain insight into additional properties at the edges of reliable TFBS, Di-nucleotide-environment views are introduced through which compositional enrichment in the flanking regions can be revealed. In combination, the software and methods allow for researchers to launch deeper explorations of TFBS within ChIP-Seq data.

The work builds on a substantial foundation of bioinformatics approaches to regulatory sequence analysis. Over-representation analysis of known TF motifs complements *de novo* motif discovery methods such as MEME. The later methods often incorporate better background representation to account for the non-random properties of sequences. The CB-plots highlight the problem for over-representation methods, providing a useful means for researchers to rapidly assess the quality of results. We introduced the BiasAway tool to correct for the bias. The HOMER 2 package includes an unpublished option for background correction of over-representation analysis, which does not perform as well as the GC composition matched option in BiasAway (HOMER was originally described in [11]). The open-access BiasAway provides a general purpose background generation capacity, which can be used as a component in other tools going forward. Both BiasAway and the CB plots are being incorporated into the online oPOSSUM-3 motif over-representation analysis tool [12].

The spatial analysis of TFBS positions within ChIP-Seq data has been a central focus in the development of several bioinformatics methods. The MEME Suite includes the CentriMo software which can evaluate both known motif collections and *de novo* pattern discovery derived motif models based on the centrality of predicted TFBS within ChIP-Seq peak regions [14]. The SpaMo component of the same package evaluates the statistical significance of spacing between predicted pairs of TFBS across ChIP-Seq regions [30]. The GEM system performs *de novo* pattern discovery of spatially correlated motif pairs [31]. While these approaches touch on the same theme, they are not directly comparable to the work presented here. Our methods are complementary to the published work, allowing researchers to more fully explore the properties of ChIP-Seq data.

We introduced the HADB procedure for the selection of appropriate PWM score thresholds for the prediction of TFBS within ChIP-Seq data. The threshold parameter for TFBS calling with PWMs is treated in diverse ways in the research literature. In some cases thresholds are individually selected for each matrix based on the determination of empirical p-values based on distributions of scores observed for a sequence pool [32]. This has been extended to generating empirical p-value thresholds for composition-matched pools of sequences [33]. In other cases, percentile-based scores are applied uniformly across diverse matrices [12]. Biophysical approaches based on the calculation of binding energies have also been introduced [34]. The HADB procedure provides an experimental basis for the selection of the threshold parameter for a TF that can be applied to the matrix in multiple data sets, as the results show little variation in the value for different ChIP-Seq studies for the same TF.

The prevalence of directly bound regions in ChIP-Seq data has received increasing attention. The ChIP-Seq protocol is undeniably successful at retrieving TF bound regions, however, the data from these studies of sequence-specific TFs is invariably shaped by a mixture of biological, experimental and computational influences. From our perspective, TF ChIP-Seq data is effectively bi-partite: a subset of ChIP’d regions have a direct relationship to the ChIP’d TF made evident by the presence of the TF’s binding motif near the peak local maximum, while the remaining subset of regions are lacking the motif. We found that on average, for the ~340 datasets studied, a third of peak regions lack canonical motifs proximal to the peakMax. Some of these peaks may result from indirect interactions (in which the ChIP’d TF interacts with a different DNA bound protein) or result from a change to the ChIP’d TF’s binding properties due to an interaction partner. Methods have been proposed to infer indirect binding by the enrichment of secondary TFs motifs, such as [35], but the presence of secondary motifs do not reliably confirm the presence of the ChIP’d TF at the regions. We have observed that the motifs of some TFs are enriched at the peakMax for more than 10 other unique TFs ChIP-Seq datasets across diverse cell lines (Worsley-Hunt, submitted), suggesting that some peaks in ChIP-Seq datasets may result from a mechanism that is not specific to the ChIP’d TF. Some additional potential sources of non-direct ChIP-Seq regions, unrelated to indirect binding, include chromatin structure properties, antibody properties, stochastic noise, or peak calling software. Two recent papers have identified highly transcribed regions as enriched in the non-direct subset of ChIP-Seq data in yeast [9, 36]. We do not suggest that any peaks be dismissed, as all are potentially informative. However, we believe that it is appropriate to use the methods introduced in this report to segregate the direct-binding subset that can be explained by the ChIP’d TFs motif within the limited range of confidence around the peakMax, allowing the two subsets to be individually analyzed.

We highlighted the investigative potential of the methods and visualization approaches in two case studies. First, a study of TBP (TATA binding protein) ChIP-Seq data, where an enrichment of SINE elements and many secondary TFs’ motifs suggests that the adaptive use of repetitive sequences as promoters may be more frequent than widely thought. Second, a study of ZNF143 that revealed the ZNF143 canonical motif is part of a wider pattern than presented by the existing PWM, which has been shown by [29] to be the binding site for THAP11, a protein that works antagonistically with ZNF143.

## Conclusion

This broad survey of ChIP-Seq data from diverse studies provides greater clarity about the properties of the data that impact the quality of interpretation. Using the methods presented here, the applied researcher can visualize the distribution of predicted TFBSs and calculate thresholds for both the maximum motif enrichment distance from the peakMax, and the PWM scores, for classifying TFBS-containing peaks. In addition to ChIP-Seq peak classification, the PWM scores thresholds set a lower limit on the range of motif scores expected for functional sites, which will be useful for downstream analyses such as binding site mutation analysis. These methods and approaches will improve TFBS enrichment analyses and the applied analysis of ChIP-Seq data, particularly for the annotation of reliable TFBSs within ChIP-Seq peaks.

## Methods

### Datasets

We downloaded ChIP-Seq datasets from the GEO database: 1) GSE11431 - thirteen mouse ES cell datasets [37]; 2) GSE25532 – mouse NFYA data in ES cells [38]; 3) GSE17917 and GSE18292 – human KLF4, POU5F1, C-MYC, NANOG, and SOX2 data [39]; and 4) GSE22078 – human and mouse C/EBPA and HNF4A [40]. A dataset for mouse FOXA2 was downloaded from http://www.bcgsc.ca/data/histone-modification/histone-modification-data [41]. We also used ENCODE ChIP-Seq datasets (human and mouse), and human ChIP-Seq controls [42] downloaded from the UCSC ENCODE database [43]. The ENCODE broadPeak datasets frequently occurred in replicate; we selected the larger replicate for our analyses. Where only the mapped sequence data was available (5 datasets), we called peaks using FindPeaks 4.0 [44] with the following parameter options: −dist_type 1 200 -subpeaks 0.6 -trim 0.2 -duplicatefilter.

As the downloaded ChIP-Seq peaks were reported with a multitude of lengths, ranging from 1 bp to >5,000 bp, we trimmed or extended all peaks to a constant length (201 bp, 401 bp, or 1001 bp) centered at the local peak maximum, or at the peak centre for datasets which do not have peakMax positions provided.

A control set of “mappable” regions was generated from the CRG Alignability (36mer) data [42] downloaded from the UCSC ENCODE database [43]. Unique, contiguous CRG Alignability 36mer regions were merged, and the resulting larger regions were then split into multiple non-overlapping regions of length 201 bp or 401 bp. This yielded two datasets of mappable regions, to be used as a source of background sequences in later analyses.

The DNase accessible control datasets used in over-representation analyses were generated from DNase I hypersensitivity datasets (UW DNase: University of Washington) [42] downloaded from the UCSC ENCODE database [43]. To obtain a dataset that reflected a broad range of DNase accessibility, DNase I hypersensitive segments from multiple cell lines were concatenated, and contiguous or overlapping regions merged. The resultant sequences were split into one of the two lengths, 201 bp and 401 bp, to generate two large datasets of DNase accessible sequences to be used as a source of background sequences in later analyses.

The Ensembl Perl API was employed to retrieve sequences from GRCh37/hg19 and NCBI37/mm9 assemblies. Where the genome coordinates first needed conversion to GRCh37/hg19 or NCBI37/mm9, we used a locally installed version of the UCSC lift-over tool [45].

Position frequency matrices (PFMs) were obtained from the JASPAR [1] development 4.0_alpha database of transcription factor models (April 2013). As the development set has been subsequently revised for a curated release, we provide the entire set of matrices as used in this report in Additional file 14. The PFMs were converted to position weight matrices (PWMs) using the TFBS Perl modules [46], with default background values for a uniform nucleotide background.

### Motif prediction

Motif prediction was performed with C code adapted from the TFBS Perl Modules [46], which scans sequences for TFBS instances and reports both the motif location and a PWM relative motif score. Where multiple motifs per sequence per PWM were predicted, the reported motifs were not permitted to overlap by more than one-fifth the PWM length (*e.g*. a 7 bp motif could only overlap a neighbouring motif by 1 bp).

### Nucleotide composition

The mononucleotide GC content of sequences was determined from a count of each nucleotide type in the sequence, based on single stranded DNA. The composition of the TF profiles was determined similarly; from a count per each nucleotide base in the position frequency matrix (PFM), and subsequently obtaining the ratio of GC nucleotides.

### Motif over-representation analyses

Motif over-representation analyses were performed on 201 bp and 401 bp sequences with a locally installed version of oPOSSUM 3.0 [12] and the online ASAP tool [47]. Within oPOSSUM we used the sequence-based analysis default settings, aside from supplying our own set of 166 PFMs. The oPOSSUM software converts the PFMs to PWMs using the default setting of the TFBS Perl modules [46]. Over-representation scores of “infinite” value were set to the greater of either 500 or to the maximum non-infinite enrichment score plus 100. For ASAP analyses, we chose parameter values similar to those used for the oPOSSUM analyses. The ASAP tool limited the number of nucleotides submitted for each analysis, therefore our input was limited to a randomly selected subset of 10,000 sequences on each submission.

The backgrounds used for the over-representation analyses were individually generated for each set of target sequences to be analyzed, and matched to the length of the target sequences. The backgrounds were derived from either the target sequences (*e.g*. dinucleotide shuffle methods, or a Markov model), a dataset of uniquely mappable sequences, or DNase accessibility data (see Datasets, above).

### Naïve backgrounds

For evaluation purposes we retained naïve backgrounds in our assessment, which were composed of randomly selected sequences from either the uniquely mappable portion of the human genome, or DNase accessible regions.

### 3^rd^ order Markov model background

A 3rd order Markov model background was generated using a combination of the oligo-analysis and random-seq programs from a local installation of the RSAT package [10]. The target sequences of interest were submitted to RSAT::oligo-analysis with the parameters: −l 4 -1str (word length 4 bp, and single strand), and the results file was in turn submitted to RSAT::random-seq with the parameter: −ol 4. The RSAT::random-seq program then used a 3rd order Markov model trained on 4 bp oligo-nucleotides to generate sequences that matched the GC content and length of the target sequences. While Markov models have been previously introduced to generate background sequences, the ideal order of the models is not clear. If the order is too high, the model will simply recapitulate the frequency of the TFBSs in the target dataset, which is why we restricted the model to 3rd order.

### HOMER 2 GC background

HOMER 2 [11] was downloaded from http://homer.salk.edu/homer/ and installed on a cluster. The findMotifsGenome.pl tool was used to perform a known motif over-representation analysis on each of the 43 datasets using the following options: −N number_of_seqs –nomotif –dumpFasta –size length_of_sequence. The number_of_sequences was the number of sequences in each dataset, and the length_of_sequence was either 201 bp or 401 bp, dependent on the analysis. The –dumpFasta option was set in order to retrieve the backgrounds after the analysis was finished running. We provided the hg19 human genome (from UCSC) and the coordinates of the peaks for each dataset.

### BiasAway Background Generating Tool

Six background models were implemented as a background sequence generator: BiasAway. The BiasAway tool has been implemented in Python using BioPython modules [48], and is available as open source code from GitHub https://github.com/wassermanlab/BiasAway/archive/noRPY.zip. The tool provides a user with six approaches for generating a background useful to over-representation analyses: 1) mononucleotide shuffled sequences, 2) dinucleotide shuffled target sequence to preserve the dinucleotide composition of the target sequences, 3) genomic sequences matched to the mononucleotide GC content of each target sequence to preserve the non-random association of nucleotides, 4) sliding windows of mononucleotide shuffled sequence, 5) sliding windows of dinucleotide shuffled target sequence, and 6) genomic sequences matched in windows of internal mononucleotide GC content for each target sequence. The latter two backgrounds (BiasAway 5–6) are variants of the former two backgrounds (BiasAway 2–3), in which we utilized a sliding window over the ChIP-Seq sequences to determine a distribution for local regions of composition. The background sequence set is then generated (dinucleotide shuffle) or selected from a pool of genomic sequences (genomic composition match) to match the distribution of window compositions for each target sequence. These latter backgrounds were considered because due to evolutionary changes such as insertion of repetitive sequences, local rearrangements, or biochemical missteps, the target sequences may have sub-regions of distinct nucleotide composition. See Additional file 1: Text S1 Supplemental Methods for greater detail.

### Measures to evaluate over-representation analysis results

To summarize the impact that the choice of background has on the reported over-representation results, we assessed the over-representation results by four measures: 1) the skew of the over-representation scores, 2) the count of datasets with the ChIP’d TF’s binding profile reported in the top 5 over-representation results, 3) the mean of the over-representation scores (excluding outlying scores), and 4) the variability of the over-representation scores (excluding outlying scores). The first measure, the skew of the over-representation scores, is the negative of the slope of the line fitted to the over-representation scores (y-value) and the associated PFM GC content values (x-value; see Figure 2a for reference). The skew informs us of the degree to which a dataset is biased towards extreme composition motifs *i.e*. GC-rich or AT-rich; the more negative the skew, the greater the bias. The second measure calculates the proportion of datasets for which the over-representation analysis (using a given background type) reports the ChIP’d TF’s motif in the top 5 results. The skew and the proportion of datasets are plotted together (Figure 2a). Background types that consistently yield a low skew value and return the ChIP’d TF in the top results, are ideal. We used the third and fourth measures to assess the tendency for the over-representation analysis to report the majority of TF’s motifs as having a low over-representation score. Therefore we set a threshold of the mean plus one standard deviation, to remove the highest over-representation scores from further analysis. We then obtained the mean and standard deviation of the remaining scores. Ideally the results would display both a low mean and a low standard deviation, as this indicates that the majority of TF’s motifs are close to an enrichment score of 0.

### Enrichment visualization plots and HADB boundaries of enrichment

For the evaluation of ChIP-Seq datasets with the HADB method, we restricted to those datasets for which we had a PWM for the ChIP’d TF.

#### Composition-Bias plots

Composition-Bias (CB) plots are generated to detect \whether GC-rich or AT-rich PWMs are over-represented in a TFBS motif over-representation analysis. The GC content of the PFM’s submitted to the over-representation analysis are presented on the x-axis, and the over-representation score of the predicted TFBSs on the y-axis.

#### TFBS-landscape view

TFBS-landscape views were generated to visualize central enrichment of a TF’s motifs within a ChIP-Seq dataset. TFBS prediction was done on 1001 bp regions, to extend sequences sufficiently far into the flanks to present the background rate of TFBS prediction. The view’s left plot presents the motif score of the top scoring predicted motif in each peak for the given TF PWM on the y-axis, and the motif’s signed distance from the peakMax on the x-axis, where the peakMax is x = 0. The right plot presents two densities depicting: 1) the distances of the top scoring motifs to the peakMax for all peaks, and 2) the distances to the peakMax for the subset of peaks for which the top scoring motif was equal to or greater than 85 to capture cases with low enrichment of strong scoring motifs. The value of 85 was based on the default parameters of the oPOSSUM software, for which 85 was chosen to best discriminate known TFBS from background. The x-axis of the right plot is the distance of the motifs to the peakMax (x = 0), and the y-axis is the probability density, which is a reflection of peak frequency for given distances. The default peak length was 1001 bp. For the TBP case study, we overlaid multiple smoothed densities of motif-to-peakMax distance enrichments on one plot (Figure 7b).

#### HADB motif enrichment threshold

The data used for the TFBS-landscape plots (TFBSs and 1001 bp sequences), was also used by the HADB method. To calculate a heuristic enrichment threshold we identified the distance from the peakMax at which the density of the top scoring motifs (one motif per sequence) falls below the density of motifs in the background regions, such as we see in the TFBS-landscape plots (Figure 4b). The background is the region spanning 200–500 bp from the peakMax. We created 5 bp bins of the absolute distance from the peakMax, and counted the number of peaks with a top scoring motif within each bin. The count in each bin was converted to the proportion of peaks in the dataset and a regression line was fitted to the background bins, where x was the upper limit of the 5 bp bin, and y was the proportion of peaks in the bin. To adjust for the variation in the y-values, we created an ‘allowance line’ by shifting the regression line along the y-axis by 2-fold the 3rd largest residual value (we omitted the two most extreme residuals as they were generally outliers). We then selected the largest non-background bin (X) that had a y-value greater than the allowance line’s predicted y-value at bin X (Figure 4a). The heuristic threshold for motif enrichment was set at the greatest distance represented by bin X (*e.g*. bin X is 100 bp-105 bp, therefore the threshold is 105 bp).

#### HADB motif score lower threshold

A similar procedure was used to determine a lower limit threshold for a PWM’s motif scores (Additional file 9: Figure S7). We generated bins of the top motif scores (one per peak) and compared the bins in the enrichment zone to bins in the background zone. Bins were populated with the count of peaks corresponding to a bin’s motif score range. Each bin’s range was 1 score point *e.g.* for bin81, the range was 80 < X < =81. ‘Central bins’ were within the enrichment zone as defined using the heuristic enrichment thresholds described above. An equal number of ‘control bins’ were generated using background regions outside of the enrichment zone, of the same width as the central enrichment zone (*e.g*. if the central enrichment zone was 180 bp, then 180 bp of background motif scores were also sampled and binned). The two sets of bins were compared to identify all the central score bins that exceeded the number of peaks in their matching control bin by greater than 20% of the maximum control bin; the maximum was selected from the set of control bins equal to or of higher value than the bin of interest (*i.e*. to the right of the bin of interest on the x-axis of Additional file 9: Figure S7a). By using the set of control bins of higher value than the bin of interest, we limited cases where low scoring bins with few peaks in the bin were selected as a threshold. The array of bins that passed the aforementioned 20% threshold was then used to determine the lower boundary of motif score enrichment. Proceeding from the upper score bins to the lower score bins, we identified the lowest set of 5 consecutive central bins (centered on the bin of interest) where at least 4 of the bins, including the bin of interest, exceeded the control bins. The latter requirement was to reduce the chance of the algorithm selecting a central bin that was an outlier in the surrounding neighbourhood of bins. The central bin preceding the flagged bin was selected as the threshold. We provide two PWM thresholds in Additional file 10: a threshold based on signal 20% above the background, as outlined above, and a threshold based on signal 5% above the background; the median absolute difference between the two different thresholds is 2 points (*e.g*. a motif score of 80 *versus* 82). The parameter of >20% signal was a heuristic limit selected to reduce stochastic noise in the HADB results.

The diversity of enrichment zones and relative motif score thresholds from the datasets were summarized in Figure 4c, Additional file 8: Figure S6a and Additional file 9: Figure S7c-d. The enrichment zone thresholds for multiple datasets of a given TF were averaged to select one threshold value per TF. To assess the similarity of the thresholds for a given TF, we calculated the average of the differences between every dataset’s threshold for the given TF, and plotted the average difference as bars above and below the average threshold. We used the same approach for reporting the average motif score and the similarity of motif scores per TF.

#### TFBS-bi-motif view

For each given sequence, the left plot reports the location of the first motif relative to the peakMax on the y-axis, and the location of the second motif relative to the first motif on the x-axis. A horizontal band of enrichment around y = 0 shows that the first motif is enriched at the peakMax, while a band of enrichment on the diagonal is the second motif’s enrichment at the peakMax. A gap at x = 0 is due to restricting the overlap of motifs. The diagonal limits of the plot arise from the sequence length limit, here 1001 bp. Increased density at the origin indicates that a number of peaks have both motif 1 and motif 2 enriched at the peakMax. The x-axis on the left plot was set as the distance between motifs in order to mirror the right plot, The right plot is a 10 bp resolution histogram of the distances between the two motifs.

#### Dinucleotide-environment view

To create a Dinucleotide-environment view, we wrote a Perl script to align a set of sequences on the motif of interest with the motif oriented in the same direction, and assess the frequency of dinucleotides at every position of the aligned sequences. The file of dinucleotide frequencies is submitted to an R script (R version 2.14.1). The R script calculates the proportion of sequences with a particular dinucleotide at each position of the alignment, and plots the position on the x-axis and the proportion of sequences on the y-axis.

Visualization was performed using the statistical package R (version 2.14.1) [13]. The R functions for generating the four visualization plots and the perl code for generating dinucleotide frequencies from motif-anchored sequence alignments are available at GitHub https://github.com/wassermanlab/TFBS_Visualization/archive/master.zip. Plots for visualizing the heuristic threshold decisions, as seen in Figure 4a and Additional file 9: Figure 7a, are included in the provided R code.

### Analysis of broadPeak replicate experiments

The ENCODE broadPeak datasets are available as replicates, which allowed us to determine whether the peaks predicted to be directly bound by the ChIP’d TF due to the presence of the TF’s motif were enriched for occurrence in both replicate experiments. Replicate experiments were pooled together, with neighbouring peaks (two peak maximums within 500 bp or 1000 bp) flagged as a single instance of a region. The 500 bp distance was selected to be inclusive of the ~400 bp median width of the broadPeak regions; 1000 bp was selected to explore the sensitivity to longer settings. Using a Fisher exact test, we then compared the proportion of replicated regions versus regions unique to a single experiment for the set of peaks with the ChIP’d TFs motif within the heuristic enrichment zone, and for the set of peaks without the ChIP’d TFs motif. Peaks that had been flagged as having the ChIP’d TFs motif in one experiment but without the ChIP’d TFs motif in the other experiment were rare (median 0.28% of the pooled replicates for an experiment), and we chose to omit these from the analysis.

### GO term enrichment analysis

We used the default settings of the web-based version of GREAT [16] http://bejerano.stanford.edu/great/public/html/splash.php. We submitted those peaks predicted to contain the ChIP’d TF’s motif by the HADB method, the peaks without the HADB predicted ChIP’d TF’s motif and peaks from the whole dataset. We used the Binomial Region Set Coverage and the number of terms related to actin and oxidative stress, to assess differences between the three sets of peaks.

### TBP case study – repeat elements analysis

Repeat elements in the TBP peaks were assessed using data downloaded from the UCSC mm9 Repeat Masker track via the UCSC Table Browser [49].

## Electronic supplementary material

Additional file 1: Text S1: Supplemental Data and Methods. (PDF 151 KB)

Additional file 2: Figure S1: Nucleotide composition is variable between ChIP-Seq datasets. **(a)** The y-axis presents the GC content of ChIP-Seq datasets (x-axis) generated from the K562 cell line; one dataset per TF. **(b)** The GC content (y-axis) of datasets from multiple samples (source cell lines indicated along the x-axis) for the JUN-D TF. (PDF 2 MB)

Additional file 3: Figure S2: The multiplicity of predicted TFBS motifs in ChIP’d sequences corresponds to the multiplicity +1 of control sequences. The number of peaks with a given multiplicity are plotted on the x-axis and the mean GC composition of the peaks is on the y-axis. ‘X’ is the motif multiplicity of the controls, and ‘X + 1’ is the motif multiplicity of the ChIP-Seq peaks. **(a)** C/EBPB ChIP-Seq sequences (black) and control sequences matching the average GC composition of the ChIP-Seq sequences (red). **(b)** AP2γ ChIP-Seq sequences (black) and control sequences matching the average GC composition of the ChIP-Seq sequences (red). (PDF 487 KB)

Additional file 4: Figure S3: Binding site over-representation results using the ASAP tool. The CB-plots present the PFM GC composition on the x-axis and the ASAP over-representation score on the y-axis. The top 5 over-represented TF profiles’ names are written on the plot; the name of the ChIP’d TF or related TF is highlighted in magenta, and the logos are shown in **(a)** E2F1 and E2F4, **(f)** JUN-family (JUN, JUN-D, AP1, and FOSL2), and **(k)** C/EBPA and CEBP/B. **(b)-(e)** E2F1 ChIP-Seq. **(g)-(j)** JUN-B ChIP-Seq. **(l)-(o)** C/EBPB ChIP-Seq. The first CB-plot for each of the 3 TFs **(b)**, **(g)**, **(l)** are results using a random background selected from a pool of uniquely mappable sequences. The second CB-plot for each of the 3 TFs **(c)**, **(h)**, **(m)** are results using a background generated by a 3rd order Markov model. The third CB-plot for each of the 3 TFs **(d)**, **(i)**, **(n)** are results using dinucleotide shuffled target sequences as background. The last CB-plot for each of the 3 TFs **(e)**, **(j)**, **(o)** are results using background sequences from the mappable dataset, matched to the GC composition distribution of the target sequences. (PDF 758 KB)

Additional file 5: Figure S4: Background impact on over-representation analyses for 400 bp datasets. **(a)** For each background, the fraction of the 43 analyses that reported the ChIP’d TF in the top 5 over-represented PWMs from a particular background (x-axis) is plotted against the average skew of the over-representation results for each background’s 43 analyses. Skew is the negative slope of the line fitted to the over-representation scores versus PFM GC content (*i.e*. values visualized by Figure 1a axes). The ideal is to have a large x-axis value (vertical dashed line) and an average skew of zero (horizontal dashed line). **(b)** and **(c)** summarize the standard deviation (y-axis) and mean (x-axis) of the ‘non-outlier’ oPOSSUM over-representation scores for 10 backgrounds against each of 43 ChIP-Seq datasets, where panel **(b)** displays the average value for each background across the 43 datasets and panel **(c)** displays the individual value of 430 analyses. The ideal result would be situated at the origin (the intersection of the dashed lines. For all panels, each of the 10 backgrounds tested is denoted as a single colour: Light green circle – randomly chosen background from the dataset of mappable sequences, dark green cross – randomly chosen background from the dataset of DNase accessible sequences, orange circle – mononucleotide shuffled background, brown cross – mononucleotide shuffled background within a sliding window, black circle – dinucleotide shuffled background, gray cross – dinucleotide shuffled background within a sliding window, magenta triangle – 3rd order Markov model generated background sequences, blue circle – background selected from the mappable sequences dataset to match the GC composition of the target sequences, light blue cross – background selected from the mappable sequences dataset to match the distribution of GC composition in sliding windows of the target sequences, and red triangle – GC background from HOMER 2. (PDF 1 MB)

Additional file 6: Table S1: Rankings of the ChIP’d TFs binding profile from over-representation analysis with 10 backgrounds. The table lists the rank of the ChIP’d TFs profile from 430 over-representation analyses for 43 datasets and 10 backgrounds. The tendency for some backgrounds to have a large bias towards TFs with GC-rich binding profiles is presented as the average skew for each background. A background with a large skew factor (>100) will favour TFs with GC-rich profiles. (PDF 71 KB)

Additional file 7: Figure S5: Background selection can correct the over-representation score bias towards GC-rich or AT-rich TFBSs in motif over-representation analyses. The results of over-representation analyses for an E2F1 ChIP-Seq dataset using six distinct backgrounds (one background per plot). The names of the 5 top ranked TF PWMs are written on the plot. The horizontal line is set at over-representation score 100 as a visual reference point. Points corresponding to E2F1 and E2F4 motifs are highlighted in pink. The dotted line at over-representation score 100 is for visual reference. The sequence logos are E2F1 and E2F4 respectively. **(a)** Randomly chosen background from a pool of DNase accessible sequences. **(b)** Randomly generated background sequences based a 3rd order Markov model. **(c)** A background of dinucleotide shuffled target sequences. **(d)** Selected regions from the mappable sequence dataset matching the GC composition distribution of the target sequence set. **(e)** Sliding windows of dinucleotide shuffled target sequence. **(f)** Genomic sequences matched in windows of internal GC composition for each target sequence. (PDF 2 MB)

Additional file 8: Figure S6: Zones of motif enrichment defined around the peakMax of mouse ChIP-Seq datasets vary per TF. **(a)** Zones of PWM motif enrichment defined by a heuristic enrichment threshold for mouse datasets. The average width of the motif enrichment zone around the peakMax for TF’s datasets are plotted on the y-axis; the differences between all widths, for all of a TF’s datasets, were averaged and plotted on the y-axis as vertical bars. The datasets are along the x-axis. The red horizontal line is the mean width of enrichment. **(b)** The proportion of peaks within the motif enrichment zone for a TF’s set of ChIP-Seq datasets were averaged. The x-axis provides, for each of 39 TFs, the mean proportion of peaks with a motif scoring above the motif score threshold and located within the zone of enrichment (mean 0.65, median 0.72). (PDF 589 KB)

Additional file 9: Figure S7: Defining a PWM’s lower bound of motif score enrichment within the heuristic enrichment zone. **(a)** A visual depiction of the motif score lower threshold determined with our heuristic procedure. The x-axis indicates the upper bound of bins of PWM motif scores for an NFYB ChIP-Seq dataset. The bins of motif scores are in steps of 1 score point (e.g. bin80 is 79 < scores < =80). The y-axis is the count of peaks in each bin. The black dotted line depicts scores for peaks with top-scoring motifs within the enrichment zone around the peakMax, while the red dotted line depicts scores for peaks with top-scoring motifs from distal positions. The vertical blue line depicts the threshold for motif score enrichment relative to the background. **(b)** The TFBS-landscape view for the NFYB dataset. The x-axis is the distance of the top scoring motif to the peakMax and the y-axis is the motif score. The blue line is the calculated motif score threshold. **(c)** and **(d)** The mean motif score threshold of multiple datasets. The motif score thresholds for a TF’s multiple datasets were averaged and plotted on the y-axis, with vertical lines showing the average differences between the thresholds. The x-axis is the TF. The red horizontal line is the mean. Left **(c)** is human data, and right **(d)** is mouse data. (PDF 2 MB)

Additional file 10: **Motif relative score thresholds for position weight matrices (PWMs).** (XLS 56 KB)

Additional file 11: Figure S8: GREAT analysis results on SRF ChIP-Seq data. GREAT results from the analyses of three sets of SRF peaks. **(a)** All peaks in the SRF dataset. **(b)** The subset of peaks identified by the HADB method to have an SRF motif proximal to the peakMax. The red box highlights the actin related GO term. **(c)** The subset of peaks that do not have an SRF motif proximal to the peakMax. (PDF 4 MB)

Additional file 12: Figure S9: GREAT analysis results on NFE2L2 ChIP-Seq data. GREAT results from the analyses of three sets of NFE2L2 peaks. **(a)** All peaks in the NFE2L2 dataset. **(b)** The subset of peaks identified by the HADB method to have an NFE2L2 motif proximal to the peakMax. The red box highlights the oxidative stress related GO terms. **(c)** The subset of peaks that do not have an NFE2L2 motif proximal to the peakMax. (PDF 3 MB)

Additional file 13: Figure S10: TFBS-landscape view of four PWMs in a TBP ChIP-Seq dataset from mouse MEL cell-line. TFBS-landscape views are shown for four PWM’s on a TBP dataset. The left-side plots present the top scoring motif distance to the peakMax on the x-axis, and the motif score on the y-axis. The right-side plots present a histogram of motif distances: black – 2 bp resolution of the top scoring motif distance per peak, green – 5 bp resolution of the distances for the top scoring motifs with a score equal to or higher than 85. Sequence logos indicate the profile used to scan the TBP peaks: **(a)** ZNF143_b PWM. **(b)** NF2F1 PWM. **(c)** CTCF PWM. **(d)** FOXD3 PWM. The data represent a subset of TF profiles depicted in Figure 7b. (PDF 2 MB)

Additional file 14: **Position frequency matrices (PFMs).** (TXT 35 KB)

## Abbreviations

ChIP-Seq: Chromatin immunoprecipitation and high-throughput sequencing
CB: Composition-bias
peakMax: Peak local maximum
PFM: Position frequency matrix
PWM: Position weight matrix
TF: Transcription factor
TFBS: Transcription factor binding site.
