# Supplementary material for: Improving analysis of transcription factor binding sites within ChIP-Seq data based on topological motif enrichment
Source: BMC Genomics. 2014 Jun 13;15(1):472. doi: 10.1186/1471-2164-15-472 (PMC4082612; doi:10.1186/1471-2164-15-472)
Supplement: Supplementary file 1 — Additional file 1: Text S1: Supplemental Data and Methods. (PDF 151 KB) [file 12864_2013_6188_MOESM1_ESM.pdf]

Supplemental Data

Sequence composition impact on TFBS prediction

Given that ChIP-Seq data displays a wide range of GC composition (Additional file 2 Figure S1), we reviewed the impact of low and high GC sequence composition on the occurence of TFBS motifs. All peaks were trimmed to 201bp prior to TFBSs prediction within the peaks. The sequences were binned by the multiplicity of the predicted TFBS over a given motif score threshold of 85. The GC composition of the bins was evaluated. For a peak region bound in a sequence-specific manner, we expect at least one TFBS for the ChIP'd TF to be present in a perfect experiment. Additional TFBS patterns may reflect function, but could arise at a frequency consistent with chance. In considering this alternative, for a multiplicity of 1 through 5 of predicted TFBSs we plotted the relatonship between peak count and mean peak GC composition. We observed a strong correspondance between control multiplcity X and peak multiplcity X+1. In other words, the ChIP-seq peaks appear to have only one extra TFBS relative to chance expectation. The Pearson correlation between ChIP-Seq peaks with multiplicity X+1 and control peaks with multiplicity X was >0.99 for the peak count, and >0.99 for the mean GC composition (Additional file 3 Figure S2).

TFBS motif over-representation analyses

The impact of background composition selection on TFBS over-representation results

As indicated in the main text, we prepared several backgrounds to be evaluated with respect to their impact on TFBS motif over-representation analyses. The first background was a naïve background model of random genomic sequences (derived from both DNase regions and from genomic mappable regions). A second background was generated by a 3rd order Markov model (RSAT package [1]). Six additional background models were implemented as a background sequence generator, BiasAway: 1) mononucleotide shuffled target sequence, 2) dinucleotide shuffled target sequence, 3) genomic sequences matched to the nucleotide composition of the target data, 4) sliding windows of mononucleotide shuffled target sequence, 5) sliding windows of dinucleotide shuffled target sequence, or 6) genomic sequences matched in sliding windows of internal composition for each target sequence. One set of backgrounds was generated by HOMER 2 [2], a tool-kit for next generation sequencing analysis. HOMER 2 is the only software of which we are aware that uses GC composition matched backgrounds for TFBS over-representation analysis.

The backgrounds were evaluated against a set of 43 human TF ChIP-Seq datasets from ENCODE (one dataset per TF, randomly chosen), and 165 PWMs (from JASPAR 4.0_alpha development database) [3], to assess the impact on over-representation scores for peakMax-centered sequences. We trimmed peaks to 201 bp, a length selection based on analyses described below, and then repeated the analyses using sequences of double the length (401 bp). We chose two over-representation software tools that accept user-defined backgrounds: oPOSSUM 3.0 [4], and ASAP [5]. All backgrounds were evaluated

with the 43 datasets on oPOSSUM, and the results of the background evaluation are summarized in Figure 2 and Additional file 5 Figure S4 (described below). The rank of the ChIP'd TF from each of 430 analyses are provided in Additional file 6 Table S1. CB-plots for the enrichment results of E2F1 ChIP-Seq data and six backgrounds are presented in Additional file 7 Figure S5. Four backgrounds were re-evaluated on three datasets for both bias and bias correction with the ASAP tool (Additional file 4 Figure S3).

We expect certain characteristics for ideal ChIP-Seq over-representation analysis results: 1) no extreme bias towards any one range of TF profile composition, which we term "skew", 2) the ChIP'd TF profile will appear amongst the top few results, 3) the majority of over-representation scores are neither over- nor under-represented (*i.e.* over-representation score mean is close to zero), and 4) the variance of the over-representation scores is low (excluding a few TFs that are outliers relative to the distribution of over-representation scores). A low variance is desired as a large variance could potentially inhibit the discovery of secondary TFs of interest. We summarize each of these ideals with respect to the six evaluated background types in Figure 2 (201 bp) and Additional file 5 Figure S4 (401 bp). Figure 2a and Additional file 5 Figure S4a present the skew of the data (the negative slope of a line fitted to the over-representation scores) and presence of the ChIP'd TF in the top 5 results on the y- and x-axis respectively. The skew is a measure of bias in the over-representation scores, while the rank of the ChIP'd TF can be considered as a measure of confidence of the over-representation results. Figure 2b-c and Additional file 5 Figure S4b-c present the average mean of the low over-representation scores and the variance of the low over-representation scores on the x- and y-axis respectively (where "low" indicates the exclusion of profiles with scores greater than one standard deviation above the mean of the entire profile set; see Methods).

Not surprisingly, use of a naïve random background for motif over-representation analysis, from either the mappable portion of the genome or DNase accessible regions, displays a systematic bias in the results towards particular groups of TFs, in almost all cases those TF's with GC-rich binding sites (*e.g.* Additional file 7 Figure S5a). Despite the bias, the ChIP'd TF is frequently one of the top-ranked TFs in both the 201bp and 401bp sequence sets; however the ranking of the remaining high scoring TFs often arises due to the dissimilarity of the composition between the target and background datasets. The remaining backgrounds reduced the bias towards any one range of TF profiles (see y-axis of Figure 2a and Additional file 5 Figure S4a). As expected for TFBS over-representation analyses, few TFs are outliers relative to the majority of scores. However, it is the GC composition matched background results (BiasAway 3 and 6, and HOMER) that most consistently returned the ChIP'd TF in the top 5 results (see x-axis of Figure 2a, and Additional file 5 Figure S4a), and resulted in the lowest values for both the mean and variation of the low over-representation scores (Figure 2b-c and Additional files 5 Figure S4b-c). While Bias-Away and HOMER GC matched backgrounds resulted in good agreement for all measurements, Bias-Away predicted the ChIP'd TF in the top 5 results in 11 percentage points (pp) greater cases for 201 bp sequences (7 pp for 401 bp sequences) compared to HOMER generated backgrounds. Relative to the GC composition matched background, both the dinucleotide shuffled background (BiasAway

2 and 5) and 3rd order Markov model background results reported the ChIP'd TF 11pp and ~27pp less frequently (respectively), and had higher means and variances of the low over-representation scores. We speculate that the 3rd order Markov model may reflect the regeneration of TFBS motifs in the background sequences. There may be cases in which datasets with extreme nucleotide composition benefit from the sliding window approach (BiasAway 3 and 6), but in our tests neither instance of the sliding window backgrounds consistently performed notably better than the simpler GC composition matched background results.

TFBS over-representation analysis with ASAP

We performed an over-representation analysis on three ChIP-Seq datasets (E2F1, JUND, and C/EBPB) with the ASAP tool to test whether over-representation score bias is platform independent. The three datasets were selected from among those that had demonstrated both an over-representation score bias and over-representation of the ChIP'd TF using oPOSSUM. The ASAP online tool limits the number of sequences to be analyzed, therefore we selected random subsets of sequences from the datasets for testing. The backgrounds for ASAP analyses were: 1) a random genomic background, 2) sequences generated by a 3rd order Markov model, 3) di-nucleotide shuffled sequences, and 4) a GC composition matched background. As is seen in Additional file 4 Figure S3, the random genomic background results in an over-representation score bias towards GC rich TFBSs, while the remaining backgrounds correct the bias. Of the non-random backgrounds, the GC matched background results reported the expected motif among the top 5 over-representation scores for 2 of the 3 datasets, while the dinucleotide shuffle results reported the expected motif for 1 of the 3 datasets, and the 3rd order Markov model results did not report the expect motif in the top 5. The ASAP results are consistent with the oPOSSUM results for both the occurrence of over-representation score bias, and the success of the corrective procedures.

TFBS-landscape plot biological interpretation

The main text describes the properties of ChIP-Seq datasets and TFBSs that are used to generate a TFBS-landscape view, which presents the peakMax proximity and motif scores of predicted TFBSs. Figure 3, presents a number of TFBS-landscape plots for which the differences in shape are notable and worthy of comment (Figure 3a-i present motif enrichment of the ChIP'd TF). The overlying commonality between all plots is the enrichment of a motif within close proximity to the peakMax (x-axis) over a range of scores (y-axis), and the presence of a band with uniform distribution of motif scores across all 1001bp. A contributing influence to the distinct patterns observed may relate to the complexity of the motif to which the TF binds. A wide TF binding pattern, such as observed for zinc-finger proteins (Figure 3d – ZNF143, 3g – NRSF/REST, or 3k – CTCF), with strong similarity to the majority of the known binding sites for the TF (*i.e.* high scoring motifs), will not be found often by chance in the genome. This is seen in the reduced number of motifs, outside the peakMax enrichment "zone", for the higher scoring range (y-axis) compared against the lower scoring range. While the lower scoring motifs may be enriched near the peakMax they also occur more frequently in the genome

by chance; thus they are seen uniformly distributed across the 1001bp sequence space (aside from enrichment at the peakMax), which results in a horizontal "band" in a number of plots (Figure 3c-d). In the three last plots, Figure 3j-l the observed enrichment is offset from the peakMax. Figure 3j, is the CTCF PWM on H3K4me3 ChIP-Seq data; CTCF is known to be enriched in proximity to nucleosomes [6] and thus the gap likely reflects a nucleosome at the peakMax, with CTCF offset to the side. Based on the nucleosome observation, it seems reasonable for CTCF motifs in RAD21 ChIP-Seq (Figure 3k), and ELK4 motifs in NELFE ChIP-Seq (Figure 3l), that here too the gap arises from the ChIP'd protein bound to the sequence at the peakMax and a TF's binding site enriched in proximity to that protein. While the cohesin complex, which RAD21 is part of, is well known to interact with CTCF [7], it is not known whether there is an interaction between ELK4 and NELFE (a subunit of the NELF complex that binds to RNA pol II).

The non-random enrichment of lower scoring motifs in the TFBS-landscape plots, as mentioned above and seen in Figure 3, suggests that while low scoring motifs may occur by chance within genomic sequence, some will be bound by the ChIP'd TF. One of the unresolved questions in TFBS prediction is at what motif score should a threshold be set for selecting a set of motifs. This is especially of interest in the computational prediction of TFBS gain/loss of function due to mutations. The TFBS landscape plots suggest a lower limit for a motif score threshold that is specific to each predictive PWM and supported by the biology of ChIP-Seq experiments.

Regions predicted by HADB to directly bind the ChIP'd TF are enriched for peaks that co-occur between replicate experiments

The HADB approach predicts regions that are bound directly by the TF, and we reasoned that if these peaks are indeed directly bound, then they should be more consistently replicated compared to peaks without the TF's direct interaction. We used those datasets for which we had replicates to assess which peaks are consistently ChIP'd. In the main text we applied a 500bp distance window between peakMax positions to classify peak regions as replicating. Here we present the same analysis using 1000bp windows. The original value was selected based on the median peak size. We selected 1000bp to ascertain if the procedure was sensitive to this parameter. We observe little difference. Using a Fisher exact test on each dataset, we determined that for the majority of datasets the peaks predicted to bind the ChIP'd TF are significantly more likely (93% of datasets produced a Fisher exact test one tailed p-value <0.001 (92% produced p-values $<1e-09$)) to be found in both replicate experiments than are the peaks without the ChIP'd TFs motif.

GO term analyses on HADB identified ChIP-Seq peaks

We performed GO enrichment analyses, using the GREAT software [8], on the subset of peaks inferred by the HADB method to be directly bound by a TF. GO enrichment analysis for TFBSs is somewhat problematic due to the diversity of processes a TF may regulate and the proximity of TFBS to the genes regulated. We therefore chose datasets

for TFs known to be highly specific for a biological process: SRF, a master regulator of the actin cytoskeleton and contractile processes [9] (5632 peaks), and NFE2L2, a key regulator of oxidative stress response [10] (1256 peaks). We submitted three sets of peaks: 1) the whole ChIP-Seq dataset, 2) peaks from the subset of regions inferred to be directly bound by the ChIP'd TF, and 3) those peaks not inferred to be directly bound. The SRF whole dataset returned GREAT results without actin cytoksleton related terms. The subset of SRF flagged by the HADB method as containing a motif for SRF returned with the first 5 terms related to actin. The remaining peaks, without the SRF motif proximal to the peakMax, did not return actin-related terms. GREAT results for SRF are provided in Additional file 11 Figure S8. The GREAT analysis with the NFE2L2 whole dataset returned two terms related to oxidative stress terms at ranks 5 and 7 in the list ("response to reactive oxygen species" and "response to oxidative stress"). The subset of HADB selected peaks returned with the same two terms at the top of the list, but with greater Bionomial Region Set Coverage values for both terms. The 3rd term in the list was "response to hydrogen peroxide", which is related to oxidative stress. GREAT results for NFE2L2 are provided in Additional file 12 Figure S9.

Supplemental Methods

Motif enrichment analyses: BiasAway Background Generating Tool

Background 1 – Mononucleotide shuffling generator
BiasAway permutes the mononucleotides of the target sequences, thus keeping the nucleotide composition of the original sequence.

Background 2 – Dinucleotide shuffling generator
BiasAway permutes the dinucleotides of the target sequences, thus keeping the nucleotide composition of the original sequence. The dinucleotide shuffle is performed by the Altschul-Erickson permutation algorithm [11], for which we adapted the Python implementation of the algorithm written by Dr. Peter Clote [12].

Background 3 – GC Mononucleotide composition matched background selector
This option of BiasAway requires as input both the target sequences and a large set of potential background sequences. The GC nucleotide composition of each target sequence is computed and sequences are assigned to bins in steps of 1% GC. The same procedure is applied to the background pool of sequences, using a separate set of 1% GC bins. Then for each target sequence in a given GC bin, BiasAway randomly selects a background sequence from the equivalent background 1% GC bin.

Background 4 – Mononucleotide shuffling within a sliding window generator
For each sequence in the input set of target sequences, BiasAway generates a background sequence by shuffling the mononucleotides within a sliding window. Formally, it slides a window of length *W* (default 100bp) with a step *S* (default 1bp) along the sequence and, within each window, shuffling the sub-sequence within the window.

Background 5 – Dinucleotide shuffling within a sliding window generator
For each sequence in the input set of target sequences, BiasAway generates a background sequence by shuffling the dinucleotides within a sliding window. Formally, it slides a window of length W (default 100bp) with a step S (default 1bp) along the sequence and, within each window, uses the Altshul-Erickson algorithm, mentioned above, to shuffle the sub-sequence within the window.

Background 6 – GC Mononucleotide composition matched background within a sliding window background selector
For this background method, BiasAway again requires as input both the target sequences and a large set of potential background sequences. For each sequence in the set of target sequences, BiasAway slides a window of length W (default 100bp) with a step S (default 1bp) to compute the corresponding %GC composition of the sequence within each window position. From the distribution of all %GC computed from the windows, the minimum N_T, the maximum X_T, the standard deviation D_T, and the coefficient of variation V_T, are computed for each target sequence. Each sequence is assigned to a GC bin (bins are in increments of 1% GC) along with the four meta values (N_T, X_T, D_T, and V_T) of each sequence. Then the average bA, and standard deviation bD of the distribution of each of the meta values, N_T, X_T, D_T, and V_T, are calculated independently for each bin of target sequences. The $bA_{N,X,D,V}$ and $bD_{N,X,D,V}$ values are associated with the given bin. The pool of background sequences are then sorted into background 1% GC bins by evaluating the four background sequence meta values (N_B, X_B, D_B, and V_B) against the $bA_{N,X,D,V}$ and $bD_{N,X,D,V}$ values of the target GC bins; if a background sequence does not satisfy the following criteria relative to one of the target bins it is removed:

$$bA_J - cov * bD_J <= val <= bA_J + cov * bD_J$$

where J={N, X, D, V}, *cov* is a coefficient of variation given as a parameter (default 2.6), *val* corresponds to a background sequence meta value {N_B, X_B, D_B, V_B} for the background sequence being analyzed, and bA_J is the average (respecting standard deviation, bD_J) of the corresponding target meta value, for the given GC bin of the target sequences (e.g. to evaluate val=N_B of a background sequence, we use bA_N and bD_N of the target bins). Finally, for each target sequence within a given GC bin B (where B is the % GC name of the bin), BiasAway randomly selects a background sequence from the equivalent background GC bin (*e.g.* if Y target sequences are in the 60% GC bin, then BiasAway randomly selects Y sequences from the 60% GC background bin).

References

1. Thomas-Chollier M, Defrance M, Medina-Rivera A, Sand O, Herrmann C, Thieffry D, Van Helden J: **RSAT 2011: regulatory sequence analysis tools.** *Nucleic Acids Res* 2011, **39:**W86–W91.

2. Heinz S, Benner C, Spann N, Bertolino E, Lin YC, Laslo P, Cheng JX, Murre C, Singh H, Glass CK: **Simple combinations of lineage-determining transcription factors prime cis-regulatory elements required for macrophage and B cell identities.** *Mol Cell* 2010, **38**(4)**:**576–589.

3. Portales-Casamar E, Thongjuea S, Kwon AT, Arenillas D, Zhao X, Valen E, Yusuf D, Lenhard B, Wasserman WW, Sandelin A: **JASPAR 2010: the greatly expanded open-access database of transcription factor binding profiles.** *Nucleic Acids Res* 2010, **38**(Database issue)**:**D105–D110.

4. Kwon AT, Arenillas DJ, Worsley Hunt R, Wasserman WW: **oPOSSUM-3: advanced analysis of regulatory motif over-representation across genes or ChIP-Seq datasets.** *G3* 2012, **2**(9)**:**987–1002.

5. Marstrand TT, Frellsen J, Moltke I, Thiim M, Valen E, Retelska D, Krogh A: **Asap: a framework for over-representation statistics for transcription factor binding sites.** *PLoS ONE* 2008, **3**(2)**:**e1623.

6. Fu Y, Sinha M, Peterson CL, Weng Z: **The insulator binding protein CTCF positions 20 nucleosomes around its binding sites across the human genome.** *PLoS Genet* 2008, **4**(7)**:**e1000138.

7. Parelho V, Hadjur S, Spivakov M, Leleu M, Sauer S, Gregson HC, Jarmuz A, Canzonetta C, Webster Z, Nesterova T, Cobb BS, Yokomori K, Dillon N, Aragon L, Fisher AG, Merkenschlager M: **Cohesins functionally associate with CTCF on mammalian chromosome arms.** *Cell* 2008, **132**(3)**:**422–433.

8. McLean CY, Bristor D, Hiller M, Clarke SL, Schaar BT, Lowe CB, Wenger AM, Bejerano G: **GREAT improves functional interpretation of cis-regulatory regions.** *Nat Biotechnol* 2010, **28**(5)**:**495–501.

9. Miano JM, Long X, Fujiwara K: **Serum response factor: master regulator of the actin cytoskeleton and contractile apparatus.** *Am J Physiol Cell Physiol* 2007, **292**(1)**:**C70–C81.

10. Singh S, Vrishni S, Singh BK, Rahman I, Kakkar P: **Nrf2-ARE stress response mechanism: a control point in oxidative stress-mediated dysfunctions and chronic inflammatory diseases.** *Free Radic Res* 2010, **44**(11)**:**1267–1288.

11. Altschul SF, Erickson BW: **Significance of nucleotide sequence alignments: a method for random sequence permutation that preserves dinucleotide and codon usage.** *Mol Biol Evol* 1985, **2**(6)**:**526–538.

12. Clote P, Ferre F, Kranakis E, Krizanc D: **Structural RNA has lower folding energy than random RNA of the same dinucleotide frequency.** *RNA* 2005, **11**(5)**:**578–591.
