# Supplementary figures and images for: Improving analysis of transcription factor binding sites within ChIP-Seq data based on topological motif enrichment
Source: BMC Genomics. 2014 Jun 13;15(1):472. doi: 10.1186/1471-2164-15-472 (PMC4082612; doi:10.1186/1471-2164-15-472)

(a)

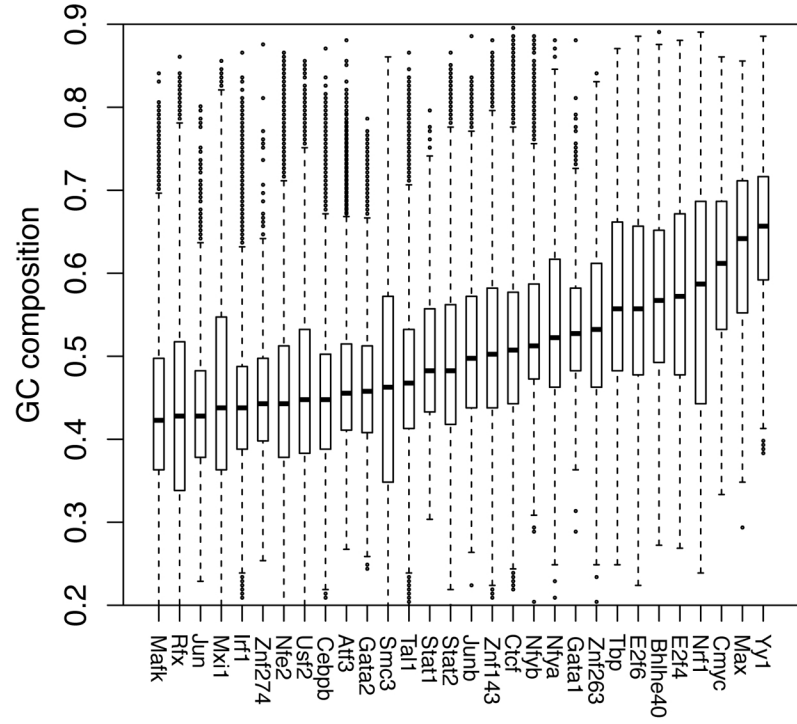

(b)

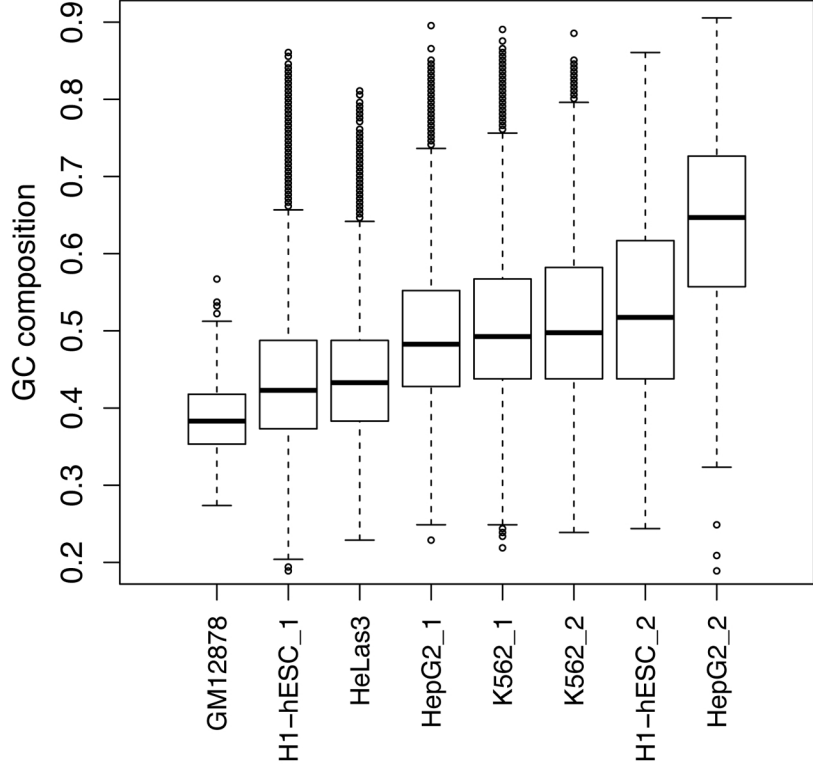

Supplement: Supplementary file 2 — Additional file 2: Figure S1: Nucleotide composition is variable between ChIP-Seq datasets. (a) The y-axis presents the GC content of ChIP-Seq datasets (x-axis) generated from the K562 cell line; one dataset per TF. (b) The GC content (y-axis) of datasets from multiple samples (source cell lines indicated along the x-axis) for the JUN-D TF. (PDF 2 MB) [file 12864_2013_6188_MOESM2_ESM.pdf]

(a)

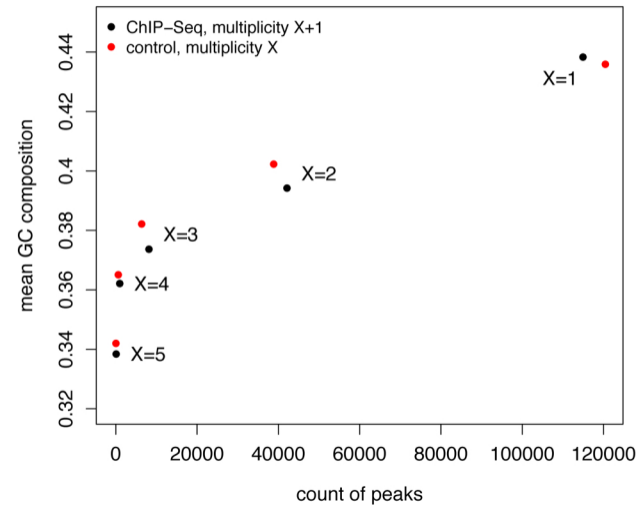

(b)

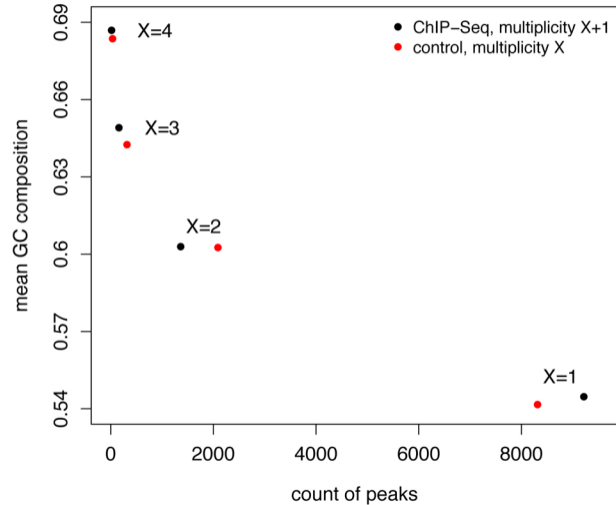

Supplement: Supplementary file 3 — Additional file 3: Figure S2: The multiplicity of predicted TFBS motifs in ChIP’d sequences corresponds to the multiplicity +1 of control sequences. The number of peaks with a given multiplicity are plotted on the x-axis and the mean GC composition of the peaks is on the y-axis. ‘X’ is the motif multiplicity of the controls, and ‘X + 1’ is the motif multiplicity of the ChIP-Seq peaks. (a) C/EBPB ChIP-Seq sequences (black) and control sequences matching the average GC composition of the ChIP-Seq sequences (red). (b) AP2γ ChIP-Seq sequences (black) and control sequences matching the average GC composition of the ChIP-Seq sequences (red). (PDF 487 KB) [file 12864_2013_6188_MOESM3_ESM.pdf]

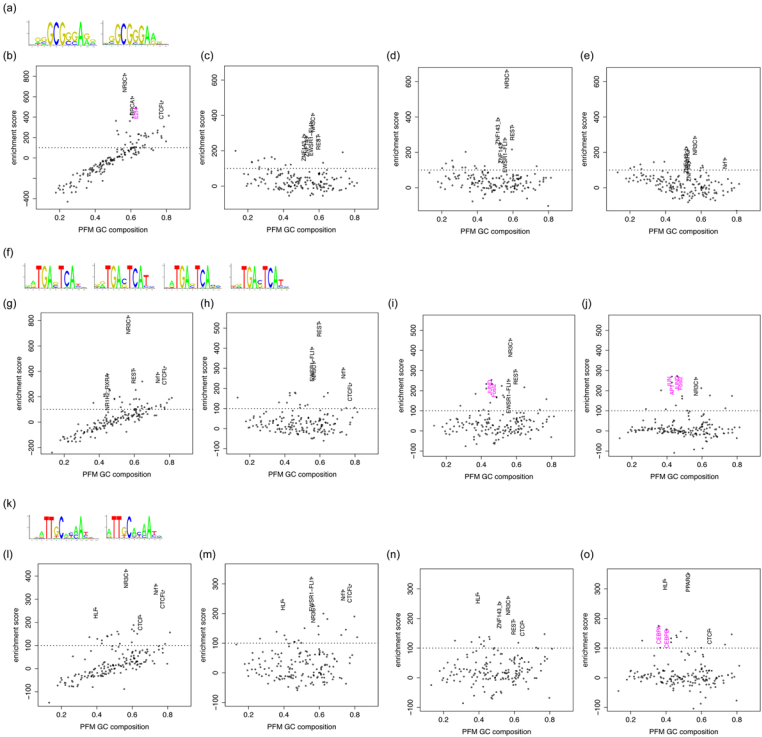

Supplement: Supplementary file 4 — Additional file 4: Figure S3: Binding site over-representation results using the ASAP tool. The CB-plots present the PFM GC composition on the x-axis and the ASAP over-representation score on the y-axis. The top 5 over-represented TF profiles’ names are written on the plot; the name of the ChIP’d TF or related TF is highlighted in magenta, and the logos are shown in (a) E2F1 and E2F4, (f) JUN-family (JUN, JUN-D, AP1, and FOSL2), and (k) C/EBPA and CEBP/B. (b)-(e) E2F1 ChIP-Seq. (g)-(j) JUN-B ChIP-Seq. (l)-(o) C/EBPB ChIP-Seq. The first CB-plot for each of the 3 TFs (b), (g), (l) are results using a random background selected from a pool of uniquely mappable sequences. The second CB-plot for each of the 3 TFs (c), (h), (m) are results using a background generated by a 3rd order Markov model. The third CB-plot for each of the 3 TFs (d), (i), (n) are results using dinucleotide shuffled target sequences as background. The last CB-plot for each of the 3 TFs (e), (j), (o) are results using background sequences from the mappable dataset, matched to the GC composition distribution of the target sequences. (PDF 758 KB) [file 12864_2013_6188_MOESM4_ESM.pdf]

(a)

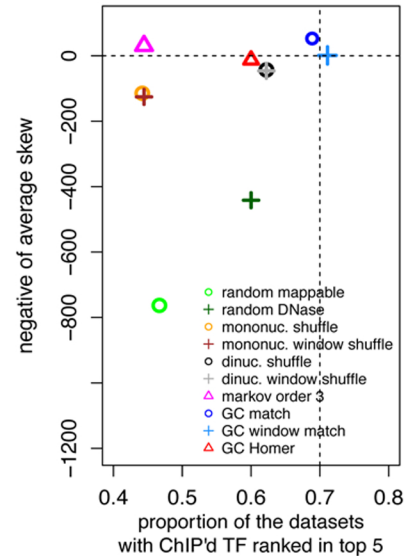

(b)

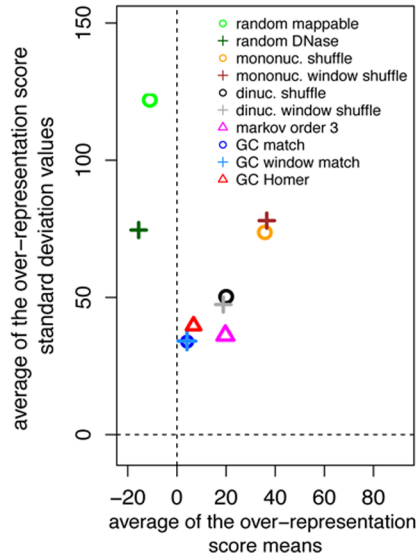

(c)

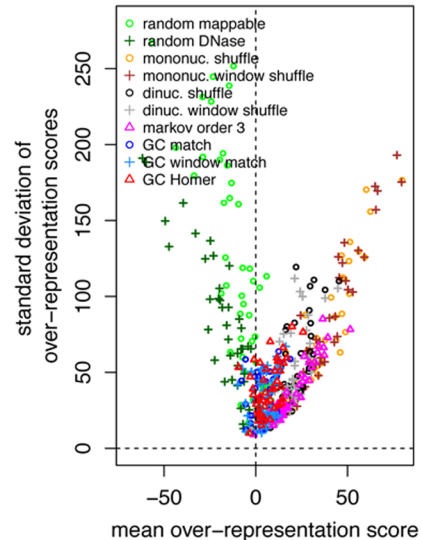

Supplement: Supplementary file 5 — Additional file 5: Figure S4: Background impact on over-representation analyses for 400 bp datasets. (a) For each background, the fraction of the 43 analyses that reported the ChIP’d TF in the top 5 over-represented PWMs from a particular background (x-axis) is plotted against the average skew of the over-representation results for each background’s 43 analyses. Skew is the negative slope of the line fitted to the over-representation scores versus PFM GC content (i.e. values visualized by Figure 1a axes). The ideal is to have a large x-axis value (vertical dashed line) and an average skew of zero (horizontal dashed line). (b) and (c) summarize the standard deviation (y-axis) and mean (x-axis) of the ‘non-outlier’ oPOSSUM over-representation scores for 10 backgrounds against each of 43 ChIP-Seq datasets, where panel (b) displays the average value for each background across the 43 datasets and panel (c) displays the individual value of 430 analyses. The ideal result would be situated at the origin (the intersection of the dashed lines. For all panels, each of the 10 backgrounds tested is denoted as a single colour: Light green circle – randomly chosen background from the dataset of mappable sequences, dark green cross – randomly chosen background from the dataset of DNase accessible sequences, orange circle – mononucleotide shuffled background, brown cross – mononucleotide shuffled background within a sliding window, black circle – dinucleotide shuffled background, gray cross – dinucleotide shuffled background within a sliding window, magenta triangle – 3rd order Markov model generated background sequences, blue circle – background selected from the mappable sequences dataset to match the GC composition of the target sequences, light blue cross – background selected from the mappable sequences dataset to match the distribution of GC composition in sliding windows of the target sequences, and red triangle – GC background from HOMER 2. (PDF 1 MB) [file 12864_2013_6188_MOESM5_ESM.pdf]

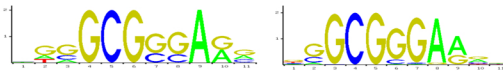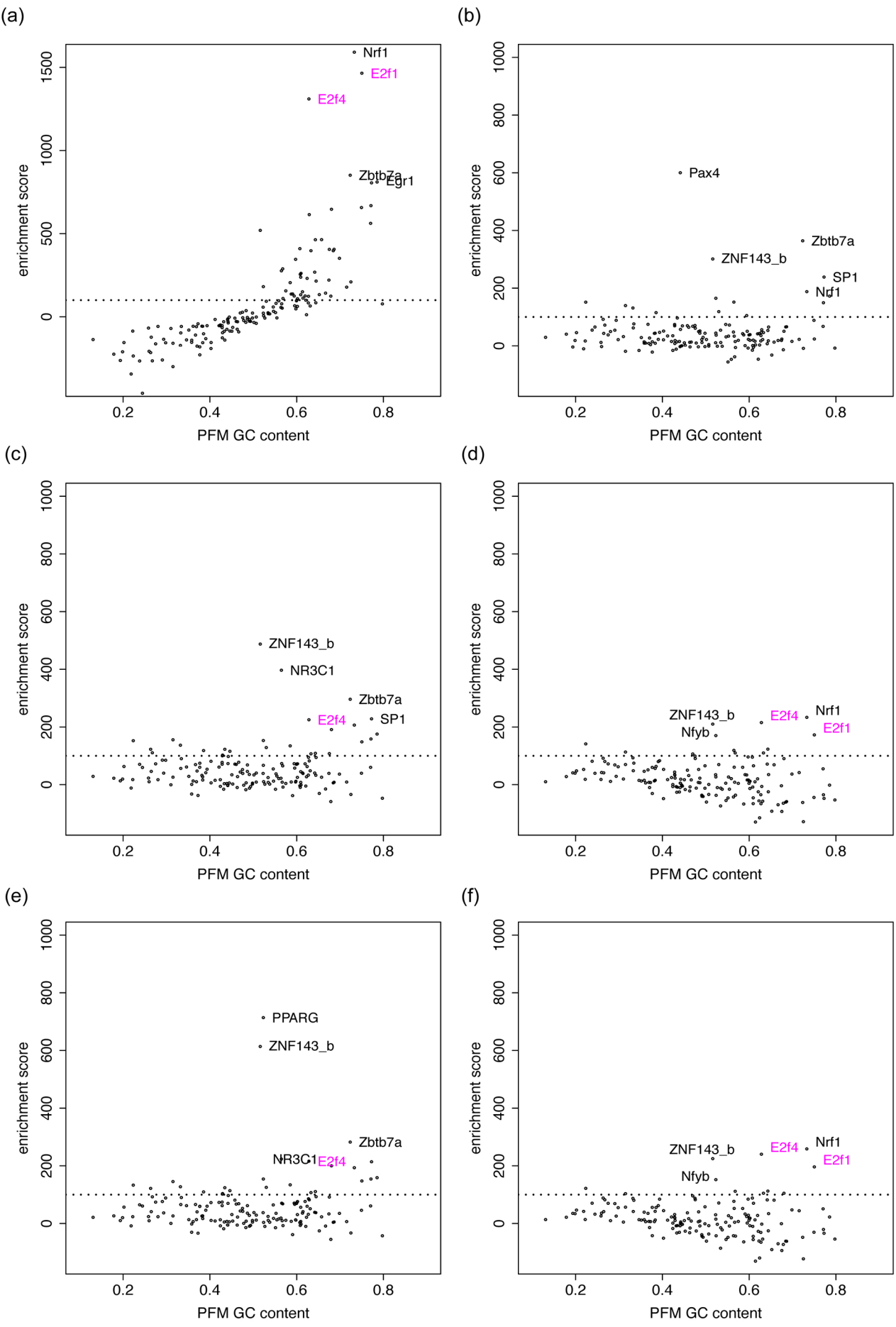

Supplement: Supplementary file 7 — Additional file 7: Figure S5: Background selection can correct the over-representation score bias towards GC-rich or AT-rich TFBSs in motif over-representation analyses. The results of over-representation analyses for an E2F1 ChIP-Seq dataset using six distinct backgrounds (one background per plot). The names of the 5 top ranked TF PWMs are written on the plot. The horizontal line is set at over-representation score 100 as a visual reference point. Points corresponding to E2F1 and E2F4 motifs are highlighted in pink. The dotted line at over-representation score 100 is for visual reference. The sequence logos are E2F1 and E2F4 respectively. (a) Randomly chosen background from a pool of DNase accessible sequences. (b) Randomly generated background sequences based a 3rd order Markov model. (c) A background of dinucleotide shuffled target sequences. (d) Selected regions from the mappable sequence dataset matching the GC composition distribution of the target sequence set. (e) Sliding windows of dinucleotide shuffled target sequence. (f) Genomic sequences matched in windows of internal GC composition for each target sequence. (PDF 2 MB) [file 12864_2013_6188_MOESM7_ESM.pdf]

(a)

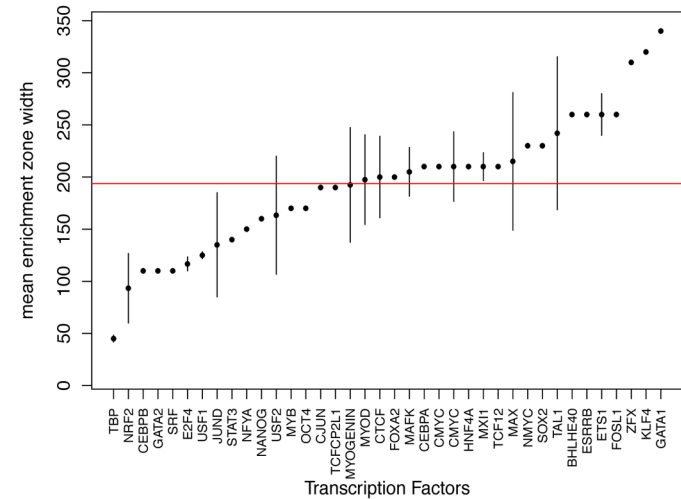

(b)

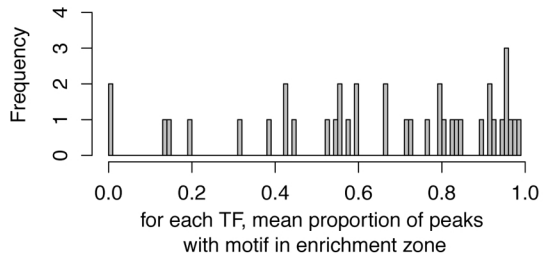

Supplement: Supplementary file 8 — Additional file 8: Figure S6: Zones of motif enrichment defined around the peakMax of mouse ChIP-Seq datasets vary per TF. (a) Zones of PWM motif enrichment defined by a heuristic enrichment threshold for mouse datasets. The average width of the motif enrichment zone around the peakMax for TF’s datasets are plotted on the y-axis; the differences between all widths, for all of a TF’s datasets, were averaged and plotted on the y-axis as vertical bars. The datasets are along the x-axis. The red horizontal line is the mean width of enrichment. (b) The proportion of peaks within the motif enrichment zone for a TF’s set of ChIP-Seq datasets were averaged. The x-axis provides, for each of 39 TFs, the mean proportion of peaks with a motif scoring above the motif score threshold and located within the zone of enrichment (mean 0.65, median 0.72). (PDF 589 KB) [file 12864_2013_6188_MOESM8_ESM.pdf]

(a)

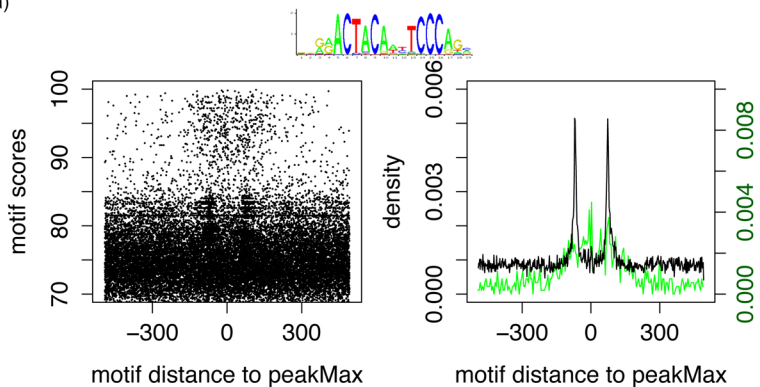

(b)

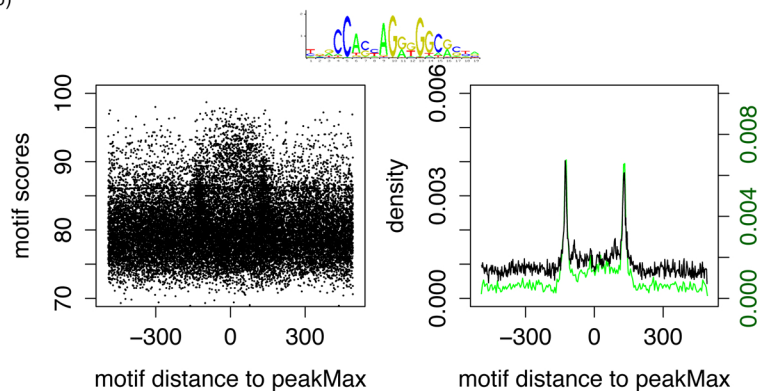

(c)

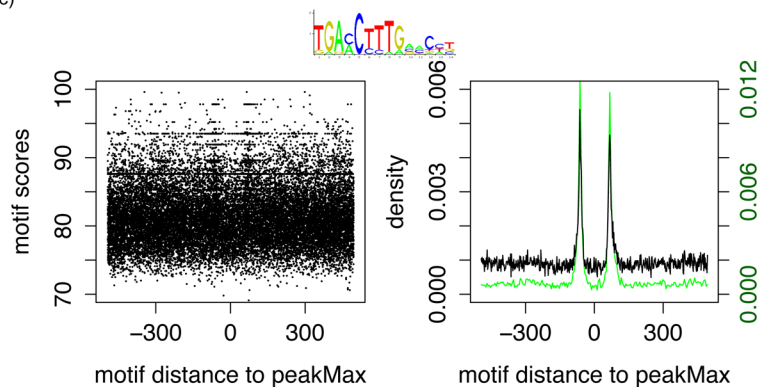

(d)

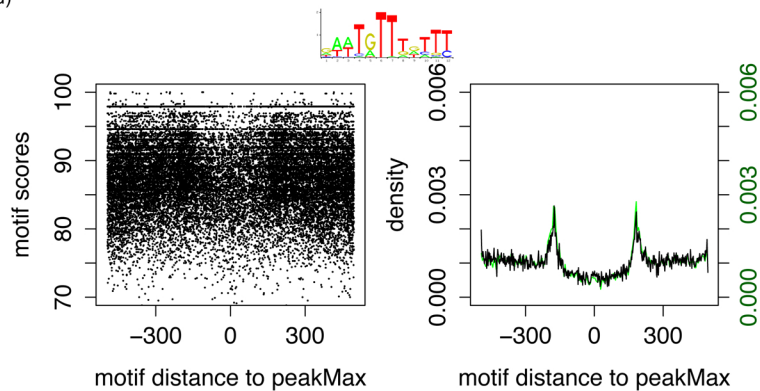

Supplement: Supplementary file 13 — Additional file 13: Figure S10: TFBS-landscape view of four PWMs in a TBP ChIP-Seq dataset from mouse MEL cell-line. TFBS-landscape views are shown for four PWM’s on a TBP dataset. The left-side plots present the top scoring motif distance to the peakMax on the x-axis, and the motif score on the y-axis. The right-side plots present a histogram of motif distances: black – 2 bp resolution of the top scoring motif distance per peak, green – 5 bp resolution of the distances for the top scoring motifs with a score equal to or higher than 85. Sequence logos indicate the profile used to scan the TBP peaks: (a) ZNF143_b PWM. (b) NF2F1 PWM. (c) CTCF PWM. (d) FOXD3 PWM. The data represent a subset of TF profiles depicted in Figure 7b. (PDF 2 MB) [file 12864_2013_6188_MOESM13_ESM.pdf]
