# Supplementary material for: Improving analysis of transcription factor binding sites within ChIP-Seq data based on topological motif enrichment
Source: BMC Genomics. 2014 Jun 13;15(1):472. doi: 10.1186/1471-2164-15-472 (PMC4082612; doi:10.1186/1471-2164-15-472)
Supplement: Supplementary file 6 — Additional file 6: Table S1: Rankings of the ChIP’d TFs binding profile from over-representation analysis with 10 backgrounds. The table lists the rank of the ChIP’d TFs profile from 430 over-representation analyses for 43 datasets and 10 backgrounds. The tendency for some backgrounds to have a large bias towards TFs with GC-rich binding profiles is presented as the average skew for each background. A background with a large skew factor (>100) will favour TFs with GC-rich profiles. (PDF 71 KB) [file 12864_2013_6188_MOESM6_ESM.pdf]

Table S1 Motif over-representation ranks for the ChIP'd TF's binding sites

	Sequence backgrounds tested in TFBS over-representation analyses									
TF	Random mappable genome	Random DNase	Mono-nucleotide shuffle	Mono-nucleotide shuffled window	Di-nucleotide shuffle	Di-nucleotide shuffled window	3rd order Markov model	GC matched	GC matched window	HOMER 2 GC matched
Average skew	617	340	118	119	41	43	-15	-18	8	13
Ap2a	2	2	5	2	2	2	3	3	2	3
Ap2g	1	1	2	4	1	2	1	1	1	2
Atf3	5	3	10	9	9	10	12	4	7	5
Bhlhe40	6	3	10	8	2	3	18	1	1	2
Brca1	4	7	70	67	18	21	22	3	3	3
Cebpb	1	1	1	1	1	1	1	1	1	1
Cfos	11	13	13	13	4	8	6	7	6	13
Cmyc	20	19	39	45	15	15	27	14	14	10
Ctcf	2	2	2	2	2	2	2	2	2	2
E2f1	2	1	13	11	13	12	83	4	4	6
E2f4	6	5	11	9	7	6	11	6	3	6
E2f6	10	6	3	5	6	6	14	16	15	19
Ebf1	1	1	1	1	2	2	3	1	1	1
Elk4	2	1	2	7	2	2	5	1	1	2
Erra	10	5	8	6	11	5	9	8	8	8
Fos	1	1	5	5	1	1	1	1	1	5
Gata1	4	6	7	7	8	6	9	4	4	6
Gata2	5	3	7	8	4	4	9	3	4	5
Gata3	10	9	6	7	7	9	21	10	13	11

Hnf4a	2	2	4	3	3	4	4	2	2	2
Hsf1	4	3	3	4	4	4	3	3	4	3
Irf1	112	119	140	151	159	161	105	124	141	114
Irf3	57	51	2	26	36	23	11	11	25	19
Junb	1	1	5	5	1	1	1	1	1	5
Jund	3	1	7	8	1	1	4	1	1	7
Jun	5	3	9	8	3	4	5	3	2	6
Max	9	5	9	10	9	9	18	9	11	10
Mxi1	22	22	36	36	18	17	44	17	17	18
Nfe2	11	5	6	6	11	13	16	13	11	12
Nfkb	3	1	4	2	1	2	1	1	1	2
Nfya	4	3	4	5	5	3	8	2	2	3
Nfyb	1	1	4	2	1	1	1	1	1	1
Nrf1	3	1	3	4	5	5	4	1	3	3
Prdm1	1	1	3	2	1	3	2	1	1	4
Rfx5	14	12	4	4	3	5	8	2	4	4
Stat1	2	1	4	5	2	1	3	1	2	1
Stat2	9	4	8	4	5	6	8	4	7	5
Stat3	120	58	22	25	48	39	161	78	105	31
Tal1	2	2	2	2	2	2	4	2	2	2
Tbp	145	123	138	148	32	31	133	35	40	52
Usf2	2	1	1	1	1	1	2	1	1	2
Yy1	2	2	4	5	2	4	5	3	2	4
Znf143	34	16	15	15	8	9	10	5	6	6
Znf263	1	1	1	1	1	1	11	1	1	1
Znf274	3	5	5	6	6	5	8	4	5	5
