# Supplementary material for: Improving analysis of transcription factor binding sites within ChIP-Seq data based on topological motif enrichment
Source: BMC Genomics. 2014 Jun 13;15(1):472. doi: 10.1186/1471-2164-15-472 (PMC4082612; doi:10.1186/1471-2164-15-472)
Supplement: Supplementary file 11 — Additional file 11: Figure S8: GREAT analysis results on SRF ChIP-Seq data. GREAT results from the analyses of three sets of SRF peaks. (a) All peaks in the SRF dataset. (b) The subset of peaks identified by the HADB method to have an SRF motif proximal to the peakMax. The red box highlights the actin related GO term. (c) The subset of peaks that do not have an SRF motif proximal to the peakMax. (PDF 4 MB) [file 12864_2013_6188_MOESM11_ESM.pdf]

(a)

Ontology	# Term Name	Binom Rank	Binom Raw P-Value	Binom FDR Q-Val	Binom Fold Enrichment	Binom Observed Region Hits	Binom Region Set Coverage	Hyper Rank	Hyper FDR Q-Val	Hyper Fold Enrichment	Hyper Observed Gene Hits	Hyper Total Genes	Hyper Gene Set Coverage
GO Biological Process	translational elongation	23	2.66872e-12	1.01655e-9	2.3947	82	1.46%	310	3.30618e-2	1.3909	60	123	0.96%
	translational termination	26	2.15818e-11	7.27225e-9	2.6314	63	1.12%	208	7.65965e-3	1.5353	49	91	0.79%
	cellular macromolecular complex disassembly	31	1.11011e-10	3.13730e-8	2.1239	90	1.60%	148	1.04368e-3	1.5300	66	123	1.06%
	macromolecular complex disassembly	34	1.49849e-10	3.86126e-8	2.1109	90	1.60%	153	1.42384e-3	1.5177	66	124	1.06%
	viral transcription	39	6.20059e-10	1.39291e-7	2.5445	57	1.01%	300	3.00110e-2	1.4748	45	87	0.72%
	positive regulation of viral transcription	50	4.92860e-9	8.63589e-7	2.5694	50	0.89%	251	1.33387e-2	1.6590	32	55	0.51%
	cellular protein complex disassembly	52	8.66704e-9	1.46023e-6	2.1161	72	1.28%	201	6.86532e-3	1.5095	54	102	0.87%
	protein complex disassembly	53	1.16800e-8	1.93073e-6	2.1000	72	1.28%	223	8.60413e-3	1.4949	54	103	0.87%
	nuclear export	62	5.29089e-8	7.47637e-6	2.1193	64	1.14%	281	2.10698e-2	1.4905	46	88	0.74%
	regulation of interferon-gamma-mediated signaling pathway	68	8.97852e-8	1.15678e-5	3.0129	32	0.57%	155	1.61958e-3	2.2315	18	23	0.29%
	RNA export from nucleus	78	2.23949e-7	2.51541e-5	2.2844	49	0.87%	311	3.35599e-2	1.5290	37	69	0.59%
	positive regulation by host of viral transcription	161	1.67836e-5	9.13301e-4	3.8039	15	0.27%	318	3.89584e-2	2.5345	8	9	0.13%
	myofibril assembly	166	2.16836e-5	1.14440e-3	2.1049	39	0.69%	317	3.89576e-2	1.7821	20	32	0.32%
	regulation of transcription from RNA polymerase III promoter	217	7.51976e-5	3.03597e-3	2.5320	23	0.41%	216	8.43560e-3	2.3167	13	16	0.21%
	blood vessel endothelial cell migration	256	1.45418e-4	4.97660e-3	2.0472	33	0.59%	283	2.12165e-2	2.1010	14	19	0.22%
	negative regulation of endocytosis	386	9.66704e-4	2.19412e-2	2.2925	19	0.34%	322	4.20793e-2	1.9960	14	20	0.22%

The test set of 5,632 genomic regions picked 6,223 genes (35%) of all 17,744 genes.	
GO Biological Process has 8,761 terms covering 14,760 (83%) of all 17,744 genes.	8,761 ontology terms were tested (100%) using an annotation count range of [1, Inf].

GREAT version 2.0.2

Species assembly: hg19

Association rule: Basal+extension: 5000 bp upstream, 1000 bp downstream, 1000000 bp max extension, curated regulatory domains included

(b)

Ontology	# Term Name	Binom Rank	Binom Raw P-Value	Binom FDR Q-Val	Binom Fold Enrichment	Binom Observed Region Hits	Binom Region Set Coverage	Hyper Rank	Hyper FDR Q-Val	Hyper Fold Enrichment	Hyper Observed Gene Hits	Hyper Total Genes	Hyper Gene Set Coverage
GO Biological Process	actin filament organization	17	3.82004e-11	1.96867e-8	2.3301	77	2.06%	285	1.36055e-2	1.6009	42	101	0.91%
	myofibril assembly	69	2.45459e-7	3.11662e-5	2.7678	34	0.91%	163	7.82467e-4	2.4062	20	32	0.43%
	ruffle organization	87	1.48668e-6	1.49710e-4	3.7778	19	0.51%	330	2.90859e-2	2.5666	10	15	0.22%
	regulation of smooth muscle cell migration	105	5.31593e-6	4.43551e-4	2.3980	34	0.91%	217	2.84570e-3	2.4639	16	25	0.35%
	actomyosin structure organization	117	7.22585e-6	5.41074e-4	2.2386	38	1.02%	190	1.42758e-3	2.1488	24	43	0.52%
	platelet-derived growth factor receptor signaling pathway	152	2.03264e-5	1.17158e-3	2.2132	35	0.94%	135	1.23653e-4	2.7719	18	25	0.39%
	regulation of metanephros development	185	6.79416e-5	3.21749e-3	2.4365	25	0.67%	318	2.43415e-2	2.4911	11	17	0.24%
	regulation of interferon-gamma-mediated signaling pathway	219	1.37295e-4	5.49241e-3	2.6982	19	0.51%	382	4.14633e-2	2.1760	13	23	0.28%
	negative regulation of endocytosis	242	1.95406e-4	7.07419e-3	2.9118	16	0.43%	352	3.33837e-2	2.3099	12	20	0.26%
	blood vessel endothelial cell migration	264	3.03812e-4	1.00822e-2	2.2457	24	0.64%	172	9.34513e-4	2.8367	14	19	0.30%
	regulation of transcription from RNA polymerase II promoter in response to oxidative stress	299	4.24146e-4	1.24279e-2	4.1468	9	0.24%	339	3.05093e-2	3.8499	5	5	0.11%
	SMAD protein signal transduction	360	8.35367e-4	2.03296e-2	2.2126	21	0.56%	281	1.27079e-2	2.6468	11	16	0.24%
	epithelial-mesenchymal cell signaling	449	1.81253e-3	3.53665e-2	2.6306	13	0.35%	371	3.93824e-2	3.2999	6	7	0.13%

The test set of 3,734 genomic regions picked 4,609 genes (26%) of all 17,744 genes.	
GO Biological Process has 8,761 terms covering 14,760 (83%) of all 17,744 genes.	8,761 ontology terms were tested (100%) using an annotation count range of [1, Inf].

GREAT version 2.0.2

Species assembly: hg19

Association rule: Basal+extension: 5000 bp upstream, 1000 bp downstream, 1000000 bp max extension, curated regulatory domains included

(c)

Ontology	# Term Name	Binom Rank	Binom Raw P-Value	Binom FDR Q-Val	Binom Fold Enrichment	Binom Observed Region Hits	Binom Region Set Coverage	Hyper Rank	Hyper FDR Q-Val	Hyper Fold Enrichment	Hyper Observed Gene Hits	Hyper Total Genes	Hyper Gene Set Coverage
GO Molecular Function	structural constituent of ribosome	1	1.21925e-10	4.14667e-7	2.8300	51	2.69%	6	7.40712e-5	2.1442	48	164	1.98%
	translation factor activity, nucleic acid binding	4	1.66816e-7	1.41836e-4	2.7125	36	1.90%	7	3.60032e-4	2.3311	35	110	1.45%
	translation elongation factor activity	7	2.04625e-6	9.94185e-4	5.2614	13	0.68%	11	3.17815e-2	3.0723	13	31	0.54%
	RNA methyltransferase activity	8	9.83230e-6	4.17996e-3	4.5357	13	0.68%	10	1.50071e-2	3.2841	13	29	0.54%

The test set of 1,898 genomic regions picked 2,422 genes (14%) of all 17,744 genes.	
GO Molecular Function has 3,401 terms covering 14,912 (84%) of all 17,744 genes.	3,401 ontology terms were tested (100%) using an annotation count range of [1, Inf].

GREAT version 2.0.2

Species assembly: hg19

Association rule: Basal+extension: 5000 bp upstream, 1000 bp downstream, 1000000 bp max extension, curated regulatory domains included
