# Supplementary material for: Improving analysis of transcription factor binding sites within ChIP-Seq data based on topological motif enrichment
Source: BMC Genomics. 2014 Jun 13;15(1):472. doi: 10.1186/1471-2164-15-472 (PMC4082612; doi:10.1186/1471-2164-15-472)
Supplement: Supplementary file 12 — Additional file 12: Figure S9: GREAT analysis results on NFE2L2 ChIP-Seq data. GREAT results from the analyses of three sets of NFE2L2 peaks. (a) All peaks in the NFE2L2 dataset. (b) The subset of peaks identified by the HADB method to have an NFE2L2 motif proximal to the peakMax. The red box highlights the oxidative stress related GO terms. (c) The subset of peaks that do not have an NFE2L2 motif proximal to the peakMax. (PDF 3 MB) [file 12864_2013_6188_MOESM12_ESM.pdf]

(a)

Ontology	# Term Name	Binom Rank	Binom Raw P-Value	Binom FDR Q-Val	Binom Fold Enrichment	Binom Observed Region Hits	Binom Region Set Coverage	Hyper Rank	Hyper FDR Q-Val	Hyper Fold Enrichment	Hyper Observed Gene Hits	Hyper Total Genes	Hyper Gene Set Coverage
GO Biological Process	positive regulation of angiogenesis	20	9.01840e-9	3.87025e-6	3.4820	30	2.39%	110	4.72228e-3	3.1077	15	61	0.94%
	response to glucocorticoid stimulus	22	1.24057e-8	4.83991e-6	2.5271	48	3.82%	147	9.00558e-3	2.1941	25	144	1.56%
	response to corticosteroid stimulus	27	1.18680e-7	3.77269e-5	2.3399	48	3.82%	189	1.65883e-2	2.0786	25	152	1.56%
	response to nutrient	29	1.35212e-7	4.00182e-5	2.0415	64	5.10%	61	6.18210e-4	2.1731	38	221	2.38%
	response to reactive oxygen species	31	1.67031e-7	4.62462e-5	2.9098	32	2.55%	184	1.50732e-2	2.3775	19	101	1.19%
	regulation of angiogenesis	32	3.01470e-7	8.08599e-5	2.5566	38	3.03%	154	9.38654e-3	2.3696	21	112	1.31%
	response to oxidative stress	35	4.98523e-7	1.22252e-4	2.1817	50	3.98%	99	3.17326e-3	2.0758	34	207	2.13%
	hepaticobiliary system development	40	1.22384e-6	2.62605e-4	2.4090	38	3.03%	205	1.98628e-2	2.4415	17	88	1.06%
	liver development	44	1.54849e-6	3.02061e-4	2.4178	37	2.95%	259	3.48927e-2	2.3513	16	86	1.00%
	response to estradiol stimulus	69	1.84744e-5	2.29806e-3	2.1843	36	2.87%	78	1.62816e-3	2.6994	22	103	1.38%

The test set of 1,256 genomic regions picked 1,600 genes (8%) of all 20,221 genes.	
GO Biological Process has 8,583 terms covering 15,210 (75%) of all 20,221 genes.	8,583 ontology terms were tested (100%) using an annotation count range of [1, Inf].

GREAT version 2.0.2
Species assembly: mm9
Association rule: Basal+extension: 5000 bp upstream, 1000 bp downstream, 1000000 bp max extension, curated regulatory domains included

(b)

Ontology	# Term Name	Binom Rank	Binom Raw P-Value	Binom FDR Q-Val	Binom Fold Enrichment	Binom Observed Region Hits	Binom Region Set Coverage	Hyper Rank	Hyper FDR Q-Val	Hyper Fold Enrichment	Hyper Observed Gene Hits	Hyper Total Genes	Hyper Gene Set Coverage
GO Biological Process	response to oxidative stress	1	1.01315e-10	8.69585e-7	3.0560	45	5.58%	32	3.08968e-4	2.5311	32	207	2.59%
	response to reactive oxygen species	2	3.96972e-10	1.70361e-6	4.1041	29	3.59%	98	3.19510e-3	2.9180	18	101	1.46%
	response to hydrogen peroxide	7	8.01526e-8	9.82785e-5	3.8449	23	2.85%	228	2.12587e-2	2.8380	13	75	1.05%
	positive regulation of angiogenesis	9	3.59556e-7	3.42896e-4	3.7936	21	2.60%	184	1.27574e-2	3.2210	12	61	0.97%
	response to inorganic substance	12	6.62364e-7	4.73756e-4	2.1094	52	6.44%	209	1.58207e-2	1.8193	36	324	2.91%
	regulation of angiogenesis	13	7.73179e-7	5.10476e-4	2.9319	28	3.47%	152	8.28756e-3	2.6314	18	112	1.46%
	cytokine production	14	1.12752e-6	6.91253e-4	3.8267	19	2.35%	229	2.18568e-2	2.9770	12	66	0.97%
	glutathione metabolic process	19	3.63045e-6	1.64001e-3	7.7470	9	1.12%	249	2.84190e-2	3.8525	8	34	0.65%
	response to estradiol stimulus	34	1.46229e-5	3.69142e-3	2.5498	27	3.35%	154	8.88330e-3	2.7024	17	103	1.38%
	response to nutrient	41	2.25081e-5	4.71189e-3	2.0355	41	5.08%	63	1.76857e-3	2.2967	31	221	2.51%

The test set of 807 genomic regions picked 1,235 genes (6%) of all 20,221 genes.	
GO Biological Process has 8,583 terms covering 15,210 (75%) of all 20,221 genes.	8,583 ontology terms were tested (100%) using an annotation count range of [1, Inf].

GREAT version 2.0.2
Species assembly: mm9
Association rule: Basal+extension: 5000 bp upstream, 1000 bp downstream, 1000000 bp max extension, curated regulatory domains included

(c)

Ontology	# Term Name	Binom Rank	Binom Raw P-Value	Binom FDR Q-Val	Binom Fold Enrichment	Binom Observed Region Hits	Binom Region Set Coverage	Hyper Rank	Hyper FDR Q-Val	Hyper Fold Enrichment	Hyper Observed Gene Hits	Hyper Total Genes	Hyper Gene Set Coverage
GO Biological Process	No results meet your chosen criteria.												

The test set of 449 genomic regions picked 494 genes (2%) of all 20,221 genes.	
GO Biological Process has 8,583 terms covering 15,210 (75%) of all 20,221 genes.	8,583 ontology terms were tested (100%) using an annotation count range of [1, Inf].

GREAT version 2.0.2
Species assembly: mm9
Association rule: Basal+extension: 5000 bp upstream, 1000 bp downstream, 1000000 bp max extension, curated regulatory domains included
