# Supplementary material for: Improving analysis of transcription factor binding sites within ChIP-Seq data based on topological motif enrichment
Source: BMC Genomics. 2014 Jun 13;15(1):472. doi: 10.1186/1471-2164-15-472 (PMC4082612; doi:10.1186/1471-2164-15-472)
Supplement: Supplementary file 9 — Additional file 9: Figure S7: Defining a PWM’s lower bound of motif score enrichment within the heuristic enrichment zone. (a) A visual depiction of the motif score lower threshold determined with our heuristic procedure. The x-axis indicates the upper bound of bins of PWM motif scores for an NFYB ChIP-Seq dataset. The bins of motif scores are in steps of 1 score point (e.g. bin80 is 79 < scores < =80). The y-axis is the count of peaks in each bin. The black dotted line depicts scores for peaks with top-scoring motifs within the enrichment zone around the peakMax, while the red dotted line depicts scores for peaks with top-scoring motifs from distal positions. The vertical blue line depicts the threshold for motif score enrichment relative to the background. (b) The TFBS-landscape view for the NFYB dataset. The x-axis is the distance of the top scoring motif to the peakMax and the y-axis is the motif score. The blue line is the calculated motif score threshold. (c) and (d) The mean motif score threshold of multiple datasets. The motif score thresholds for a TF’s multiple datasets were averaged and plotted on the y-axis, with vertical lines showing the average differences between the thresholds. The x-axis is the TF. The red horizontal line is the mean. Left (c) is human data, and right (d) is mouse data. (PDF 2 MB) [file 12864_2013_6188_MOESM9_ESM.pdf]

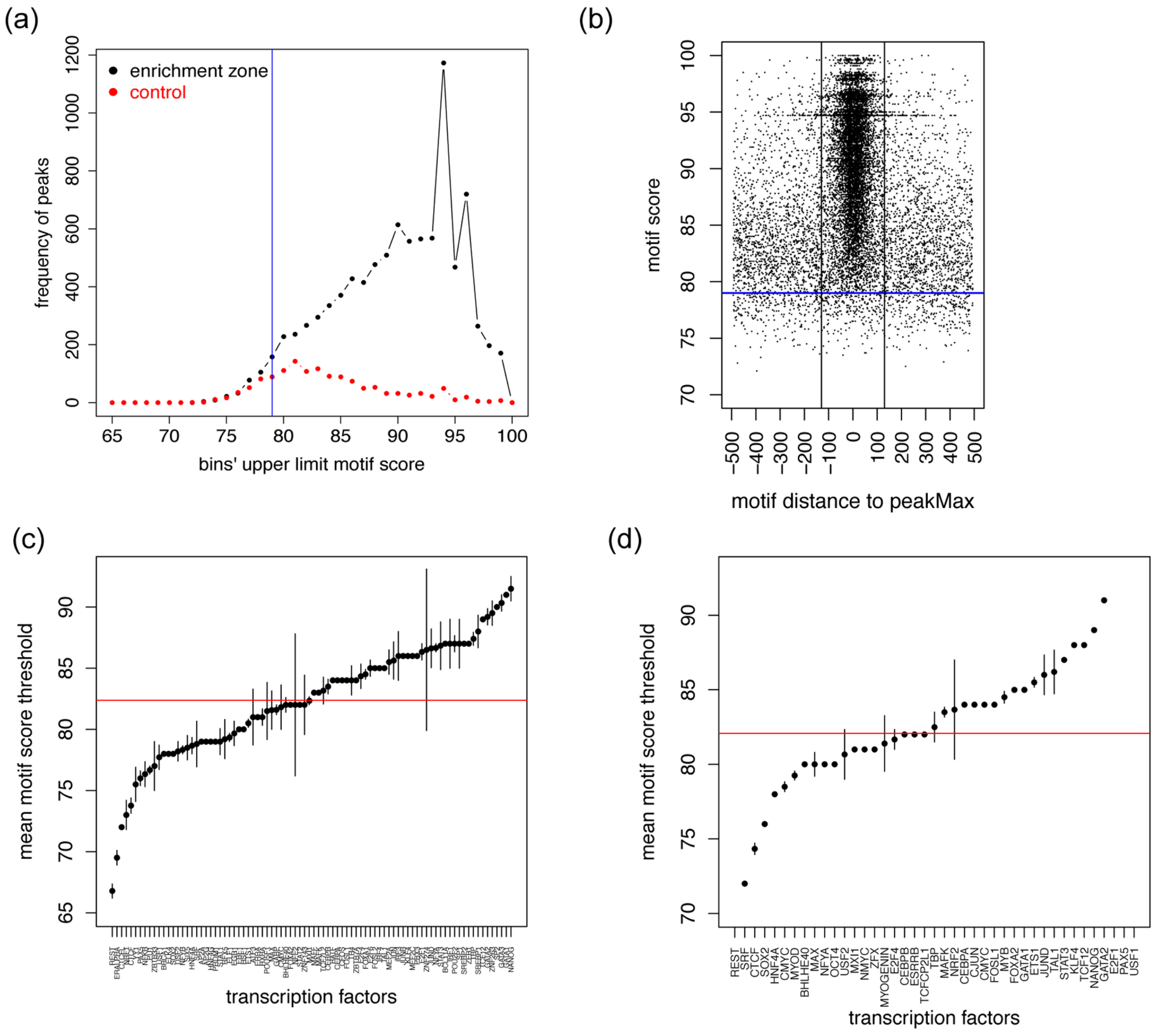

(a)
enrichment zone
control
frequency of peaks
bins' upper limit motif score
(b)
motif score
motif distance to peakMax
(c)
mean motif score threshold
transcription factors
(d)
REST
SRF
CTCF
SOX2
HNF4A
CMYC
MYOD
BHLHE40
MAX
NFYA
OCT4
USF2
MXI1
NMYC
ZFX
MYOGENIN
E2F4
CEBPB
ESRRB
TCFCP2L1
TBP
MAFK
NRF2
CEBPA
CJUN
CMYC
FOSL1
MYB
FOXA2
GATA1
ETS1
JUND
TAL1
STAT3
KLF4
TCF12
NANOG
GATA2
E2F1
PAX5
USF1
mean motif score threshold
transcription factors
